# Dorsal hyperintensity and iron deposition patterns in the substantia nigra of Parkinson’s disease, idiopathic REM sleep behavior disorder, and Parkinson-plus syndromes at 7T MRI: a prospective diagnostic study

**DOI:** 10.1186/s40035-025-00495-4

**Published:** 2025-07-04

**Authors:** Dongning Su, Zhijin Zhang, Zhe Zhang, Rui Yan, Wanlin Zhu, Ning Wei, Yue Suo, Xinyao Liu, Ying Jiang, Lingyan Ma, Huiqing Zhao, Zhan Wang, Xuemei Wang, Huizi Ma, Xin Liu, Chaodong Wang, Zhirong Wan, Fangfei Li, Yuan Li, Joyce S. T. Lam, Junhong Zhou, Ning Zhang, Tao Wu, Jing Jing, Tao Feng

**Affiliations:** 1 Department of Neurology, Beijing Tiantan Hospital, Capital Medical University, Beijing, China; 2 China National Clinical Research Center for Neurological Diseases, Beijing, China; 3 Tiantan Neuroimaging Center of Excellence, Beijing Tiantan Hospital, Capital Medical University, Beijing, China; 4 Department of Neurology, National Clinical Research Center for Geriatric Disorders, Xuanwu Hospital, Capital Medical University, Beijing, China; 5 Department of Neurology, Aerospace Center Hospital, Beijing, China; 6 Department of Neurology, Beijing Chaoyang Hospital, Capital Medical University, Beijing, China; 7 MR Research Collaboration Team, Siemens Healthineers, Beijing, China; 8 Pacific Parkinson’s Research Centre, Djavad Mowafaghian Centre for Brain Health, University of British Columbia, Vancouver, BC Canada; 9 Hinda and Arthur Marcus Institute for Aging Research, Hebrew SeniorLife, Roslindale, MA USA; 10 Harvard Medical School, Boston, MA USA; 11 Department of Neuropsychiatry and Behavioral Neurology and Clinical Psychology, Beijing Tiantan Hospital, Capital Medical University, Beijing, China

**Keywords:** 7T MRI, Substantia nigra, Dorsal nigral hyperintensity, Iron deposition, Parkinson’s disease, Idiopathic rapid eye movement sleep behavior disorder, Parkinson-plus syndromes

## Abstract

**Background:**

Dorsal nigral hyperintensity (DNH) abnormality associated with excessive iron deposition in the substantia nigra, is recognized as an imaging characteristic of Parkinson’s disease (PD) and can be effectively visualized using 7T MRI. This study was aimed to develop and validate the optimal DNH assessment method as a biomarker for PD, idiopathic rapid eye movement sleep behavior disorder (iRBD), and Parkinson-plus syndromes, and to explore the nigral iron deposition patterns in these diseases.

**Methods:**

Three-dimensional gradient-echo T2*-weighted images were acquired by 7T MRI from a total of 402 patients and 100 healthy controls (HCs) in two independent cohorts (development and validation cohorts). Seven methods, including four dichotomous methods and three DNH rating scales, were used to assess DNH and evaluate their diagnostic performance. R2* mapping and principal component analysis were performed to assess nigral iron deposition patterns.

**Results:**

Bilateral DNH detection rates in the development cohort were 22.6% for early-stage PD, 3.7% for advanced PD, 93.5% for iRBD, 5.7% for MSA-parkinsonian type, 78.8% for MSA-cerebellar type, 11.8% for progressive supranuclear palsy (PSP), and 100% for HC, with similar rates in the validation cohort. A cut-off of 6 on the 6-point visibility scale demonstrated a 100% accuracy for diagnosing early-stage PD in both the development and the validation cohorts. This scale exhibited moderate differential diagnostic performance between early-stage PD and iRBD (area under the curve [AUC] = 0.940) or MSA-C (AUC = 0.892). Iron deposition was predominantly in the dorsal and posterior substantia nigra of PD and PSP, the intermediate and posterior substantia nigra of MSA-P, and the ventral substantia nigra of MSA-C.

**Conclusion:**

DNH may be preserved in approximately one-quarter of early-stage PD and most MSA-C cases. The 6-point visibility scale on 7T effectively distinguished PD from HC, iRBD, and MSA-C. The nigral iron deposition pattern in PD may help distinguish PD from MSA-P and MSA-C, although it overlaps with that of PSP.

**Supplementary Information:**

The online version contains supplementary material available at 10.1186/s40035-025-00495-4.

## Introduction

It is challenging to diagnose Parkinson’s disease (PD) accurately, especially in the prodromal and early stages, and to differentiate PD from Parkinson-plus syndromes. Changes in the dorsal nigral hyperintensity (DNH) on iron-sensitive magnetic resonance imaging (MRI) have been recognized as an imaging characteristic of PD [1]. DNH represents or partially overlaps with nigrosome 1, which is composed of densely packed dopaminergic neurons in the substantia nigra pars compacta (SNpc) [2, 3]. Its formation may be associated with differences in iron content between nigrosome 1 and the surrounding matrix. In PD, a significant increase in cellular iron within the dopaminergic neurons in nigrosome 1 results in a more uniform distribution of iron between nigrosome 1 and the surrounding matrix [4], leading to blurred boundaries and reduced DNH intensity. Thus, alterations of the DNH and nigral iron deposition patterns could be useful as biomarkers for PD diagnosis.

Given that the R2* value of iron-rich brain tissue increases with higher magnetic field intensity, 7T MRI outperforms 3 T in iron-sensitive imaging [5] and has been employed to assess iron content in the substantia nigra and delineate the DNH [6, 7]. Previous 7T studies with small sample sizes have reported an absence of DNH in 83% to 100% of PD [8, 9]. Reduction of the 7T T2* signal in nigrosome 1 has been found to positively correlate with disease severity, suggesting progressive iron deposition and degeneration of nigrosome 1 in PD [6]. Whether the DNH is completely invisible or is only partially reduced in early-stage or prodromal PD remains to be investigated. Discrepancies in imaging parameters, such as spatial resolution and echo time, along with variations in DNH assessment methods (including dichotomous evaluation and semi-quantitative scales) [6, 10–13], may also contribute to the inconsistent results. Moreover, the utility of DNH abnormality in distinguishing PD from Parkinson-plus syndromes remains largely unexplored. The relatively small sample sizes, 10–36 for PD [2, 14] and no more than 7 for multiple system atrophy (MSA) and progressive supranuclear palsy (PSP) [15, 16], in existing 7T studies have further limited the robustness of findings using DNH as a diagnostic biomarker. Given that pathological iron deposition in substantia nigra of PD or Parkinson-plus syndromes is not confined to nigrosome 1 [17–19], analyzing the pathological nigral iron deposition patterns in PD, MSA, PSP, and idiopathic rapid eye movement sleep behavior disorder (iRBD) is warranted.

In this prospective diagnostic study, we utilized 7T three-dimensional (3D) T2*-weighted MRI to investigate the DNH abnormality and nigral iron deposition patterns in PD, iRBD, MSA-parkinsonian type (MSA-P), MSA-cerebellar type (MSA-C), and PSP. Two independent cohorts were recruited, comprising a total of 502 participants, representing the largest and most comprehensive investigation of the DNH to date. In the development cohort comprising PD, iRBD, MSA-P, MSA-C, PSP, and healthy controls (HC), DNH was evaluated using four dichotomous methods and three DNH rating scales, to determine the optimal assessment method and establish cut-off values for disease diagnosis and differential diagnosis. The validation cohort, consisting of early-stage PD, MSA-P, MSA-C, and HC, was used to confirm the results from the development cohort. Additionally, data from both cohorts were pooled, and R2* mapping and voxel-based principal component analysis (PCA) were performed to explore the patterns of nigral iron deposition across different disease groups.

## Methods

### Study design and participants

For the development cohort, all PD, iRBD, MSA-P, MSA-C, and PSP patients as well as HCs were recruited from Beijing Tiantan Hospital, China. For the validation cohort, patients with early-stage PD, MSA-P, and MSA-C as well as HCs were recruited from four centers in China. All clinical assessments and MRI scans were performed at Beijing Tiantan Hospital.

All diagnoses were confirmed by two movement disorders specialists. PD (age at onset ≥ 40) and PSP were diagnosed according to the Movement Disorder Society (MDS) clinical diagnostic criteria [20, 21], while MSA was diagnosed following the 2008 Gilman criteria [22]. iRBD patients were diagnosed based on the International Classification of Sleep Disorders, 3rd edition [23] with video polysomnography confirmation. Patients with evidence of movement disorders or dementia were excluded. PD patients were further classified into early-stage PD (disease duration ≤ 5 years and modified Hoehn and Yahr [H-Y] stage at the OFF state ≤ 2.5) and advanced PD (disease duration > 5 years and/or modified H-Y stage at the OFF state > 2.5). HCs without evidence of neurological disorder were recruited from the community. The exclusion criteria were: (1) secondary parkinsonism due to head trauma or exposure to dopamine receptor blocking agents, or vascular parkinsonism; (2) prior neurosurgery for PD or Parkinson-plus syndromes; (3) history of other neurological conditions (cerebrovascular disease, epilepsy, inflammatory diseases, brain tumor, etc.) or severe chronic underlying medical illness; (4) history of a psychiatric disorder; (5) contraindications for MRI (ferromagnetic or non-static metal implants, claustrophobia, mechanical or electromagnetic implants, or tattoos with metal ink); and (6) having severe tremor/dyskinesia or motion artifacts during MRI scan.

### Clinical assessments

Motor assessments were performed at the OFF state after an overnight washout of anti-parkinsonian drugs for at least 12 h. All patients were assessed according to the MDS-Unified Parkinson’s Disease Rating Scale Part III (MDS-UPDRS III). Mini-Mental State Examination, the Montreal Cognitive Assessment, the Hamilton Depression Rating Scale, and the Hamilton Anxiety Rating Scale assessments were performed for all participants.

### Imaging protocol

High-resolution 7T T2*-weighted images were obtained using a 3D multi-echo gradient-echo sequence. The parameters used were as follows: echo time 1 = 8 ms, echo time 2 = 15 ms, echo time 3 = 22 ms, and echo time 4 = 29 ms, repetition time = 42 ms, flip angle = 16°, spatial resolution 0.4 × 0.4 × 0.5 mm^3^, 88 axial slices, bandwidth = 160 Hz/pixel, and acquisition time = 8 min 24 s. Other MRI scans included 7T T1-weighted imaging and 3 T T1- and T2-weighted imaging. MRI scan was performed at the ON state to minimize motion artifacts. Details of image acquisition are reported in Additional file 1: eMethods.

### Visual assessment of the DNH

The presence of DNH was defined as a hyperintense ovoid substructure within the dorsal hypointense SNpc region on at least three serial planes of oblique axial T2*-weighted images from the level of the inferior part of the red nucleus to the inferior part of the substantia nigra [24]. The DNH was assessed using seven methods, including being dichotomously rated as “non-pathological” or “pathological” by bilaterally detected, unilaterally detected, bilaterally normal, and unilaterally normal methods, as well as evaluated by a 6-point visibility scale, by the area of hyperintensity using a 2-point scale, and by a 2-point swallow tail sign scale [2, 6, 11–13, 15, 25]. Detailed assessment methods are described in Additional file 1: eMethods.

All images were independently reviewed by two neurologists with over 5 years of experience in brain MRI, and discrepancies in interpretations were resolved by a third neurologist with over 15 years of experience in brain MRI.

### Image postprocessing

**Fig. 1:**
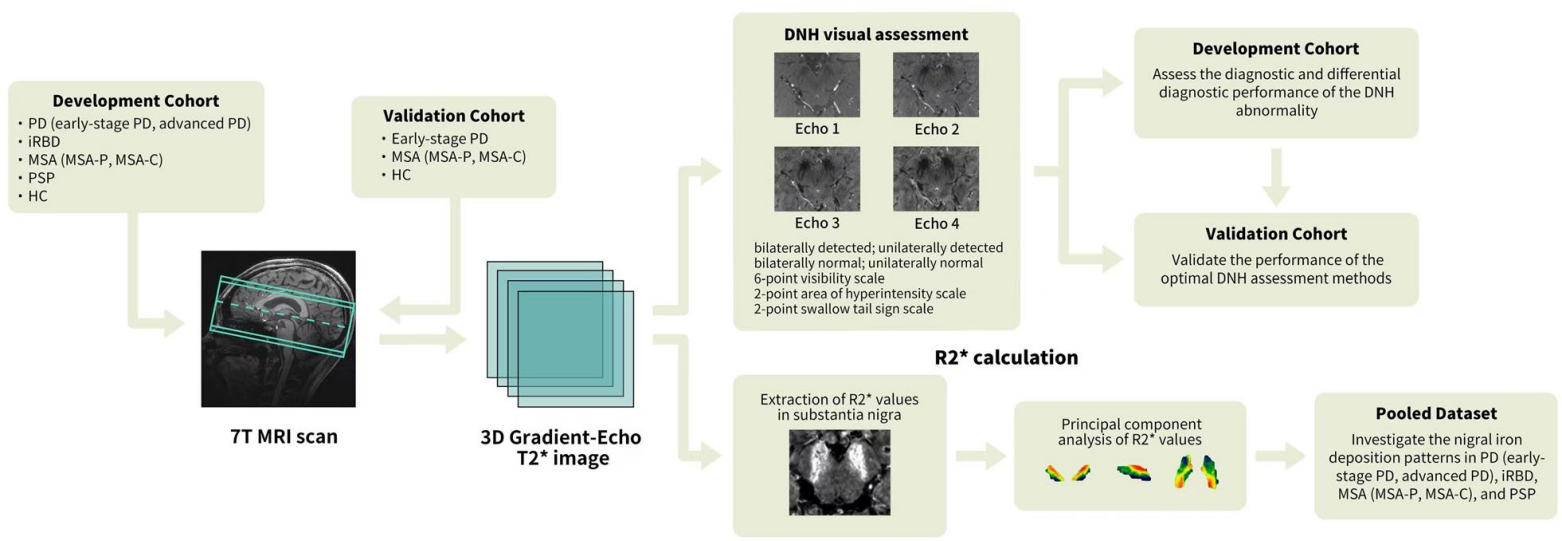
Flowchart of image assessment procedures. First, all participants underwent 7T MRI scanning. On the 3D gradient-echo T2*-weighted images acquired, DNH abnormality was evaluated using seven DNH assessment methods. A development cohort was recruited to assess the diagnostic and differential diagnostic accuracy of DNH abnormality and determine the optimal DNH assessment method. A validation cohort was recruited to confirm these results. Lastly, an R2* map was calculated and principal component analysis of the substantia nigra was performed in the pooled dataset to explore the nigral iron deposition pattern. PD, Parkinson’s disease; iRBD, idiopathic rapid eye movement sleep behavior disorder; MSA, multiple system atrophy; MSA-P, MSA-parkinsonian type; MSA-C, MSA-cerebellar type; PSP, progressive supranuclear palsy; HC, healthy control; DNH, dorsal nigral hyperintensity

T2*-weighted images in MNI space were generated using the ANTs toolbox 8 (https://github.com/ANTsX/ANTs) [26], and the R2* map was calculated according to the 4-echo T2*-weighted images in MNI space using a linear regression model with the SEPIA toolbox 9 (https://github.com/kschan0214/sepia) [27]. Voxel-wise PCA was performed on a 0.5-mm MNI-space nigral R2* map. Comparisons between all disease groups and the HC group were performed using the pooled dataset. Based on the “atlasing of the basal ganglia (ATAG)” dataset (https://www.nitrc.org/projects/atag/) [28], a mask of the substantia nigra was generated using MIAKAT (www.miakat.org) from the 0.5-mm MNI-space R2* map. Principal components (PCs) were identified based on the Akaike information criterion (AIC) model, which was applied to determine the optimal combination of PCs that explained the most significant variance in the data. Details of R2* mapping and PCA of the substantia nigra are provided in Additional file 1: eMethods. The whole assessment procedures are presented in Fig. 1.

### Statistical analysis

Statistical analysis was performed using SPSS (version 29.0, IBM, Chicago, IL). Normality of numeric variables was assessed using the Shapiro–Wilk test. Given the distribution of the numeric variables, Mann–Whitney U test or Kruskal–Wallis test with Bonferroni correction for multiple comparisons was performed. Categorical variables were compared using Chi-square test or Fisher’s exact test, with Bonferroni correction applied for multiple comparisons. Inter-observer reliability for DNH evaluation was measured using Cohen’s kappa coefficient or weighted Cohen’s kappa coefficient. The receiver operating characteristic (ROC) curve analysis with Youden’s index was used to determine the optimal cut-off value for DNH rating scales. The area under the curve (AUC) was analyzed by the DeLong’s test to assess the discriminative power of the ROC model. Diagnostic sensitivity, specificity, accuracy, positive predictive value, negative predictive value, positive likelihood ratio (LR+), and negative likelihood ratio (LR-) were calculated for all seven DNH assessment methods. LRs were interpreted as follows: LR+  > 10 and LR- < 0.1 (large probability); LR+  = 5–10 and LR- = 0.1–0.2 (moderate); LR+  = 2–5 and LR- = 0.2–0.5 (small); and LR+  < 2 and LR- > 0.5 (poor) [29]. DNH assessment methods with LR+  ≥ 5 and LR- ≤ 0.2 were compared for accuracy. Specifically, dichotomous methods with higher accuracy (Chi-square test) and rating scales with higher AUC (DeLong’s test) were compared. Spearman’s correlation was used to test associations between DNH scores and clinical features. False discovery rate was used for multiple comparison correction in PCA. *P* < 0.05 was considered statistically significant.

## Results

### Demographic and clinical data

Between June 2021 and April 2024, 300 patients and 64 HCs were recruited in the development cohort. The validation cohort comprising 121 patients and 41 HCs was recruited from four centers between May 2024 and December 2024. After excluding 24 subjects due to motion artifacts, a total of 502 participants were included in the final analysis (Tables 1 and S1, Fig. S1). Details of the between-group comparisons are presented in Additional file 1: Analysis S1.

**Table 1:** Demographic and clinical characteristics in the development and the validation cohorts

	Development cohort							Validation cohort			
	Early-stage PD	Advanced PD	iRBD	MSA-P	MSA-C	PSP	HC	Early-stage PD	MSA-P	MSA-C	HC
	*n* = 62	*n* = 54	*n* = 31	*n* = 53	*n* = 52	*n* = 34	*n* = 60	*n* = 46	*n* = 34	*n* = 36	*n* = 40
Age, year, median (IQR)	59.0 (53.0–65.0)	65.0 (59.0–68.3)	64.0 (58.0–68.0)	58.0 (54.0–66.0)	58.0 (53.0–63.8)	68.5 (62.8–72.0)	60.0 (54.0–66.0)	59.0 (50.0–64.0)	61.0 (56.3–64.5)	59.0 (53.5–65.0)	59.0 (55.0–65.5)
Sex, male/female	29/33	29/25	20/11	21/32	28/24	22/12	26/34	26/20	14/20	17/19	15/25
Education, year, median (IQR)	12.0 (9.0–15.0)	12.0 (9.0–15.8)	12.0 (9.0–15.8)	9.0 (9.0–12.0)	11.0 (9.0–12.0)	9.0 (7.5–12.0)	12.0 (9.0–16.0)	12.0 (9.0–16.0)	12.0 (9.0–12.0)	9.0 (9.0–12.0)	12.0 (9.0–15.5)
Disease duration, year, median (IQR)	2.0 (1.0–3.0)	8.0 (6.0–10.0)	3.0 (1.0–5.0)	2.0 (1.0–3.5)	2.0 (1.0–3.0)	2.0 (2.0–4.3)	–	2.0 (1.0–3.0)	2.0 (1.5–3.1)	2.0 (1.0–3.0)	–
MDS-UPDRS III (OFF), median (IQR)	26 (18–32)	40 (32–53)	2 (1–5)	40 (33–53)	25 (20–33)	38 (29–51)	–	21 (14–31)	46 (34–61)	23 (15–31)	–
H-Y stage (OFF), median (IQR)	2.0 (2.0–2.5)	3.0 (2.5–3.0)	–	–	–	–	–	2.0 (1.5–2.0)	–	–	–
MMSE, median (IQR)	28 (26–29)	28 (26–29)	29 (27–29)	28 (26–29)	27 (26–28)	24 (22–27)	29 (28–30)	29 (26–30)	28 (25–29)	28 (27–29)	29 (28–29)
MoCA, median (IQR)	23 (19–27)	22 (19–26)	25 (22–27)	23 (20–26)	23 (19–25)	19 (15–22)	26 (22–27)	24 (21–27)	23 (19–27)	22 (19–25)	24 (22–27)
HAMA, median (IQR)	8 (3–10)	7 (4–12)	8 (3–11)	7 (4–11)	6 (4–9)	6 (4–11)	4 (1–6)	6 (4–9)	9 (6–11)	9 (6–13)	4 (2–6)
HAMD, median (IQR)	6 (3–10)	6 (3–9)	7 (3–10)	7 (5–13)	7 (3–11)	8 (5–12)	4 (1–7)	8 (3–12)	9 (4–13)	7 (5–14)	3 (1–6)

PD, Parkinson’s disease; iRBD, idiopathic rapid eye movement sleep behavior disorder; MSA, multiple system atrophy; MSA-P, MSA-parkinsonian type; MSA-C, MSA-cerebellar type; PSP, progressive supranuclear palsy; HC, healthy control; MDS-UPDRS, Movement Disorder Society-Unified Parkinson’s Disease Rating Scale; H-Y, Hoehn and Yahr; MMSE, Mini-Mental State Examination; MoCA, Montreal Cognitive Assessment; HAMD, Hamilton Depression Rating Scale; HAMA, Hamilton Anxiety Rating Scale; *n*, number; IQR, interquartile range

### DNH in different T2* echoes

**Fig. 2:**
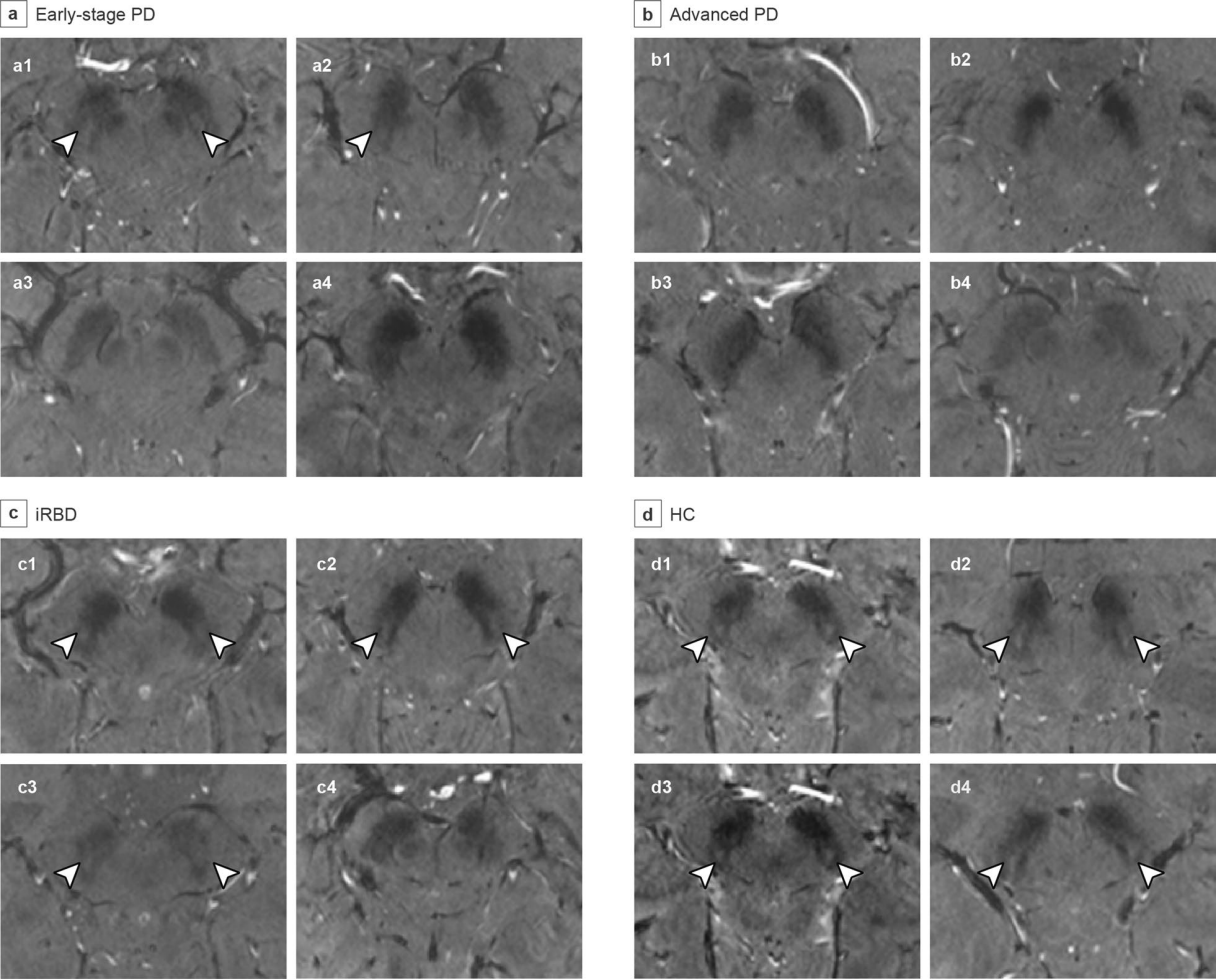
7T 3D gradient-echo T2*-weighted images of the substantia nigra in the PD, iRBD, and HC groups. 7T 3D gradient-echo T2*-weighted images at echo 2 of the substantia nigra show heterogeneity of DNH abnormality in early-stage PD (**a**) and iRBD (**c**), absence of DNH in advanced PD (**b**), and presence of DNH in HCs (**d**). Arrowheads indicate detectable DNH. DNH was detected in **a1**, **a2**, **c1–c3**, and **d1**-**d4**, and was absent in **a3**, **a4**, **b1-b4**, and **c4**. PD, Parkinson’s disease; iRBD, idiopathic rapid eye movement sleep behavior disorder; HC, healthy control; DNH, dorsal nigral hyperintensity

**Fig. 3:**
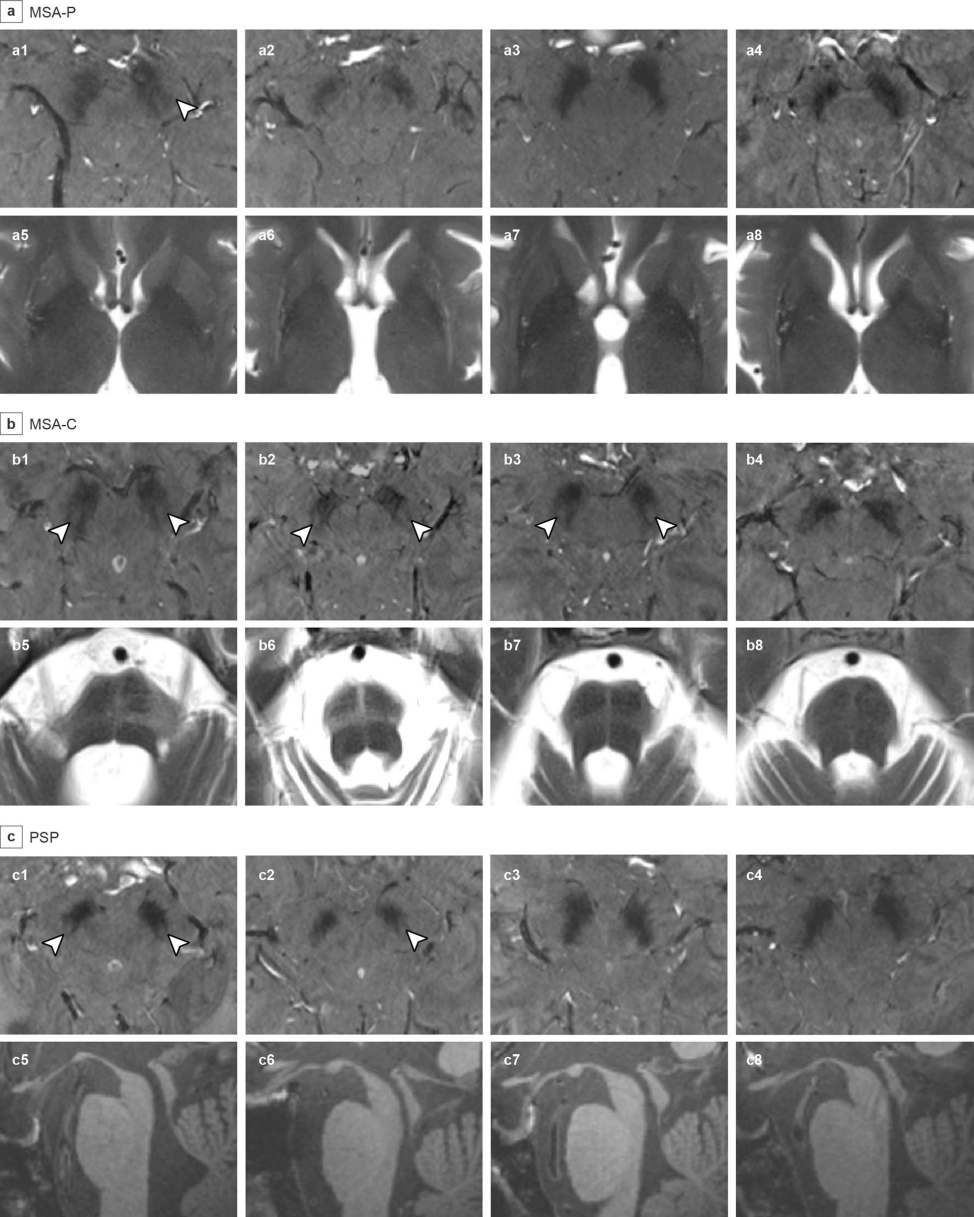
7T 3D gradient-echo T2*-weighted images at echo 2 of the substantia nigra and corresponding 3 T T1/T2-weighted images in MSA-P (**a**), MSA-C (**b**), and PSP (**c**). Arrowheads indicate detection of DNH. DNH was detected in **a1**, **b1**-**b3**, and **c1**-**c2**, and absent in **a2**-**a4**, **b4**, and **c3**-**c4**, indicating the heterogeneity of DNH abnormality in MSA-C. Corresponding axial T2-weighted images of the basal ganglia show atrophy in the putamen of MSA-P patients (**a5–a8**). Axial T2-weighted images of the pons show the hot cross bun sign in MSA-C patients (**b5–b8**). Sagittal T1 images reveal midbrain atrophy and the hummingbird sign in PSP patients (**c5–c8**). MSA, multiple system atrophy; MSA-P, MSA-parkinsonian type; MSA-C, MSA-cerebellar type; PSP, progressive supranuclear palsy; 3D, three-dimensional; DNH, dorsal nigral hyperintensity

Inter-observer reliability of the seven DNH assessment methods is presented in Additional file 1: Table S2. T2* images at four echo times from a representative case in each group are shown in Additional file 1: Fig. S2. As echo 2 generally showed better diagnostic or differential diagnostic accuracy compared with other echoes (Tables S3 and S4, Analysis S2), it was selected for further comparison of the diagnostic performance between different DNH assessment methods. Figures 2 and 3 show the T2* images of the substantia nigra in patients and HCs at echo 2. The ROC curves of the three DNH rating scales at echo 2 derived from the pooled dataset are presented in Additional file 1: Fig. S3.

### Comparison of the DNH between PD and HC, PD and Parkinson-plus syndromes

The DNH was bilaterally detected in 22.6% of early-stage PD and 3.7% of advanced PD in the development cohort, and in 17.4% of early-stage PD in the validation cohort. In the pooled dataset, the detection rate was 14.8% for PD overall and 20.4% for early-stage PD. All HCs exhibited bilateral detection of DNH (Tables 2 and S4).

**Table 2:** Visual assessment of the DNH in the development and the validation cohorts at echo 2

	Development cohort							Validation cohort			
	Early-stage PD	Advanced PD	iRBD	MSA-P	MSA-C	PSP	HC	Early-stage PD	MSA-P	MSA-C	HC
	*n* = 62	*n* = 54	*n* = 31	*n* = 53	*n* = 52	*n* = 34	*n* = 60	*n* = 46	*n* = 34	*n* = 36	*n* = 40
Bilaterally detected, *n* (%)	14 (22.6) ^a,b,c^	2 (3.7) ^a,b,c^	29 (93.5)	3 (5.7) ^a,b,c^	41 (78.8) ^a^	4 (11.8) ^a,b,c^	60 (100)	8 (17.4) ^a,c^	2 (5.9) ^a,c^	29 (80.6) ^a^	40 (100)
Unilaterally detected, *n* (%)	23 (37.1) ^a,b,c^	7 (13.0) ^a,b,c^	29 (93.5)	9 (17.0) ^a,b,c^	45 (86.5)	5 (14.7) ^a,b,c^	60 (100)	14 (30.4) ^a,c^	5 (14.7) ^a,c^	31 (86.1)	40 (100)
Bilaterally normal, *n* (%)	0 (0) ^a,b^	0 (0) ^a,b^	5 (16.1) ^a^	0 (0) ^a^	4 (7.7) ^a^	1 (2.9) ^a^	41 (68.3)	0 (0) ^a^	0 (0) ^a^	3 (8.3) ^a^	25 (62.5)
Unilaterally normal, *n* (%)	0 (0) ^a,b,c^	0 (0) ^a, b,c^	8 (25.8) ^a^	0 (0) ^a,b,c^	10 (19.2) ^a^	1 (2.9) ^a^	50 (83.3)	0 (0) ^a,c^	0 (0) ^a,c^	6 (22.2) ^a^	33 (82.5)
6-point visibility scale, median (IQR)	2 (2–4) ^a,b,c^	2 (2–2)^a,b,c^	7 (6–9)	2 (2–2) ^a,b,c^	7 (5–8) ^a^	2 (2–2) ^a,b,c^	10 (9–10)	2 (2–4) ^a,c^	2 (2–2) ^a,c^	7 (5–8) ^a^	10 (9–10)
2-point area of hyperintensity scale, median (IQR)	0 (0–0) ^a,b,c^	0 (0–0)^a,b,c^	1 (0–3) ^a^	0 (0–0) ^a,b,c^	1 (0–2) ^a^	0 (0–0) ^a,b,c^	4 (3–4)	0 (0–0) ^a,c^	0 (0–0) ^a,c^	1 (0–2) ^a^	4 (3–4)
2-point swallow tail sign scale, median (IQR)	0 (0–1) ^a,b,c^	0 (0–0)^a,b,c^	2 (2–3)	0 (0–0) ^a,b,c^	2 (2–2) ^a^	0 (0–0) ^a,b,c^	4 (3–4)	0 (0–1) ^a,c^	0 (0–0) ^a,c^	2 (2–2) ^a^	4 (3–4)

^a^ *P* < 0.05 compared with HC

^b^* P* < 0.05 compared with iRBD

^c^ *P* < 0.05 compared with MSA-C

Chi-squared test or Fisher’s exact test, with Bonferroni correction applied for multiple comparisons, was used to compare dichotomous evaluation methods. Kruskal–Wallis test with Bonferroni correction was used to compare DNH rating scale scores

PD, Parkinson’s disease; iRBD, idiopathic rapid eye movement sleep behavior disorder; MSA, multiple system atrophy; MSA-P, MSA-parkinsonian type; MSA-C, MSA-cerebellar type; PSP, progressive supranuclear palsy; HC, healthy control; *n*, number; IQR, interquartile range

**Fig. 4:**
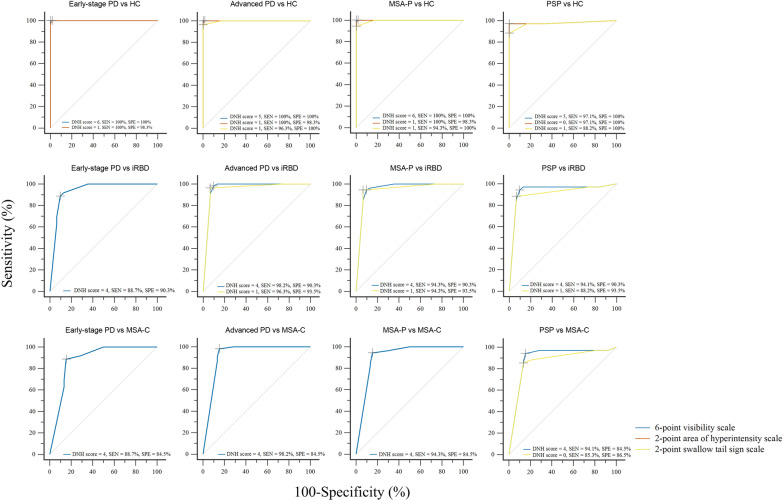
ROC curves for the optimal DNH rating scales in the development cohort. PD, Parkinson’s disease; iRBD, idiopathic rapid eye movement sleep behavior disorder; MSA, multiple system atrophy; MSA-P, MSA-parkinsonian type; MSA-C, MSA-cerebellar type; PSP, progressive supranuclear palsy; HC, healthy control; DNH, dorsal nigral hyperintensity; SEN, sensitivity; SPE, specificity

**Fig. 5:**
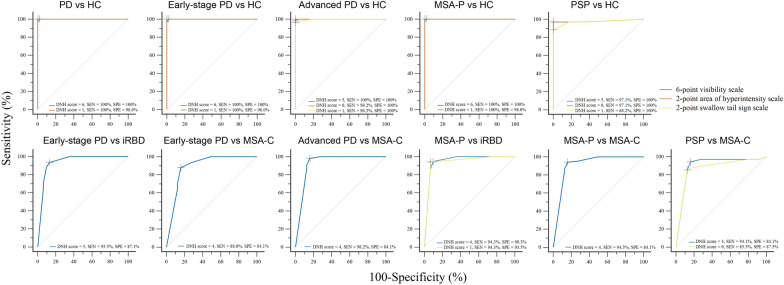
ROC curves for the optimal DNH rating scales in the pooled dataset. PD, Parkinson’s disease; iRBD, idiopathic rapid eye movement sleep behavior disorder; MSA, multiple system atrophy; MSA-P, MSA-parkinsonian type; MSA-C, MSA-cerebellar type; PSP, progressive supranuclear palsy; HC, healthy control; DNH, dorsal nigral hyperintensity; SEN, sensitivity; SPE, specificity

Compared with HCs, PD showed significantly lower percentages of detectable and “non-pathological” DNH and lower DNH scores across all seven assessment methods (Tables 2 and S4). No significant correlations were found between DNH scores and clinical features among the 44 PD patients with detectable DNH. There were also no differences in DNH assessed by all seven assessment methods between tremor-dominant and non-tremor-dominant early-stage PD in the pooled dataset (Additional file 1: Analysis S3, Table S5). In the pooled dataset, 84.0% (21/25) of PD patients with unilateral detectable DNH exhibited a contralateral correlation between the predominant side of DNH impairment and the predominant side of motor symptoms (Table S6). A cut-off score of 6 on the 6-point visibility scale achieved 100% sensitivity and specificity (AUC = 1.000) for differentiating early-stage PD from HCs in both the development and the validation cohorts. It also outperformed all dichotomous methods and the 2-point swallow tail sign scale (Figs. 4, 5; Additional file 1: Tables S7 and S9, Fig. S4).

Compared with MSA-C, early-stage PD patients showed significantly lower percentages of bilaterally detected, unilaterally detected, and unilaterally normal DNH, along with lower DNH rating scale scores (Tables 2 and S4). A cut-off score of 4 on the 6-point visibility scale demonstrated 86.8% and 85.4% accuracy in the development and validation cohorts, respectively, with pooled sensitivity of 88.0% and specificity of 84.1% (AUC = 0.892), indicating moderate discriminative performance (Figs. 4, 5 and S4, Tables S7–S9). No significant differences were observed among early-stage PD, advanced PD, MSA-P, and PSP across the seven methods (Tables 2 and S4).

### Comparison of DNH between iRBD and HC as well as early-stage PD

We identified bilateral detectable DNH in 93.5% of iRBD patients. Compared with HCs, iRBD had significantly lower percentages of “non-pathological” DNH and 2-point area of hyperintensity scale scores (Table 2).

Compared with early-stage PD, iRBD showed significantly higher percentages of detectable and “non-pathological” DNH, along with higher DNH scores across all seven assessment methods (Tables 2 and S4). The 6-point visibility scale showed moderate performance in distinguishing early-stage PD from iRBD, with AUCs of 0.937 (development cohort) and 0.940 (pooled dataset). A cut-off score of 5 yielded 93.5% sensitivity and 87.1% specificity in the pooled dataset (Figs. 4, 5 and S4; Tables S7 and S8).

### Comparison of DNH abnormality among MSA-P, MSA-C, and PSP

DNH was bilaterally detected in 5.7% of MSA-P, 78.8% of MSA-C, and 11.8% of PSP patients in the development cohort, and in 5.9% of MSA-P and 80.6% of MSA-C patients in the validation cohort. Pooled bilateral detection rates were 5.7% for MSA-P and 79.5% for MSA-C (Tables 2 and S4).

MSA-P and PSP patients showed significantly lower percentages of detectable and “non-pathological” DNH and lower DNH scores than HCs (Additional file 1: Analysis S4). MSA-C had significantly lower percentages of bilaterally detected, bilaterally normal, and unilaterally normal DNH, as well as lower DNH scores than HCs (Tables 2 and S4). No significant correlation was observed between DNH scores and clinical features in the 76 MSA-C patients with detectable DNH (Additional file 1: Analysis S3). Compared with MSA-C, MSA-P showed significantly lower percentages of bilaterally detected, unilaterally detected, unilaterally normal DNH, as well as lower DNH scores. PSP showed significantly lower bilateral and unilateral DNH detection rates and DNH scores compared with MSA-C, with no significant difference between MSA-P and PSP (Tables 2 and S4).

A cut-off score of 6 on the 6-point visibility scale achieved 100% sensitivity and specificity (AUC = 1.000) for differentiating MSA-P from HCs in both the development and the validation cohorts. The unilaterally normal method and 2-point swallow tail sign scale exhibited accuracy of 82.1% in the development cohort and 81.4% in the pooled dataset, though no method achieved moderate diagnostic accuracy for MSA-C vs HCs. For PSP vs HCs, both the 6-point visibility and the 2-point hyperintensity scales showed 97.1% sensitivity and 100% specificity in the development and pooled datasets (Figs. 4 and 5; Tables S7–S9, Fig. S4).

For differentiating MSA-P from MSA-C, a cut-off of 4 on the 6-point visibility scale showed moderate performance, with 89.5% and 88.6% accuracy in the development and validation cohorts, respectively; pooled sensitivity and specificity were 94.3% and 84.1% (AUC = 0.913). For PSP vs MSA-C, the 6-point visibility scale achieved 88.4% accuracy in the development cohort, with 94.1% sensitivity and 84.1% specificity in the pooled dataset (AUC = 0.900) (Figs. 4 and 5; Tables S7–S9, Fig. S4).

In addition, subgroup analyses among advanced PD, MSA-P, MSA-C, PSP, and HCs aged over 60 with matched age and years of education yielded results consistent with those from the development and the pooled datasets (Table S10).

### PCA of iron deposition in the substantia nigra

**Fig. 6:**
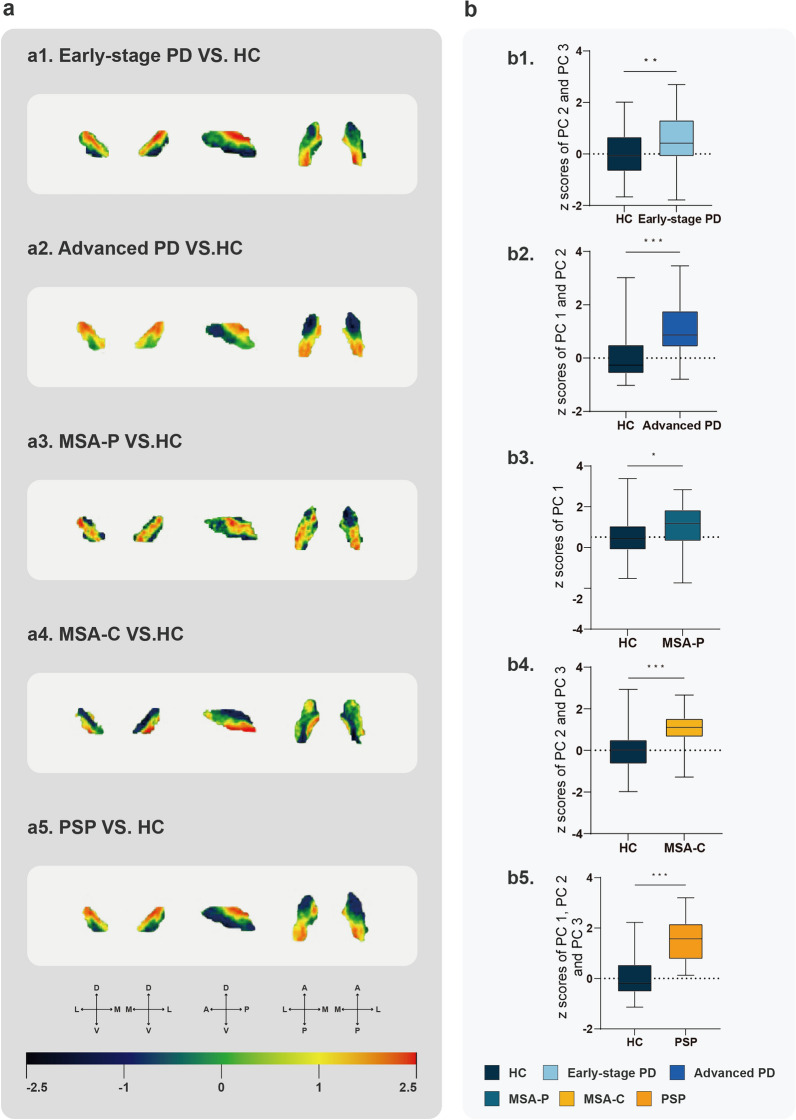
Principal component analysis of R2* values of the substantia nigra. **a** Regional weights of the R2* component(s) in the early-stage PD (**a1**), advanced PD (**a2**), MSA-P (**a3**), MSA-C (**a4**), and PSP (**a5**) groups compared with HCs in the pooled dataset. **b** Comparison of z-scores of principal component(s) of early-stage PD (**b1**), advanced PD (**b2**), MSA-P (**b3**), MSA-C (**b4**), or PSP (**b5**) with HCs. *P < 0.05; **P < 0.01; ***P < 0.0001 (false discovery rate applied for multiple comparison correction). PD, Parkinson’s disease; MSA, multiple system atrophy; MSA-P, MSA-parkinsonian type; MSA-C, MSA-cerebellar type; PSP, progressive supranuclear palsy; HC, healthy control; M, medial; L, lateral; D, dorsal; V, ventral; A, anterior; P, posterior

PCA was performed on nigral R2* maps, and comparisons were made between the disease groups and the HC group in the pooled dataset. According to the AIC model, PCs 2 and 3 accounted for 18.0% of the variance and effectively separated between the early-stage PD and HC’s pattern subject scores (*P* = 0.001426; Fig. 6b1). PCs 1 and 2 accounted for 32.9% of the variance, resulting a clear separation between advanced PD and HC’s pattern subject scores (*P* < 0.000001; Fig. 6b2). PC 1 accounted for 27.1% of the variance and effectively distinguished MSA-P from HC’s pattern subject scores (*P* = 0.012855; Fig. 6b3). PCs 2 and 3 accounted for 17.1% of the variance and effectively separated between MSA-C and HC’s pattern subject scores (*P* = 0.00005; Fig. 6b4). PCs 1, 2, and 3 accounted for 43.9% of the variance and effectively differentiated PSP from HC’s pattern subject scores (*P* < 0.000001; Fig. 6b5). Lastly, PCs 1, 2, and 3 varied across group comparisons, indicating distinct PCs.

Compared with HCs, early-stage PD, advanced PD, and PSP all exhibited higher R2* values in the dorsal and posterior substantia nigra. MSA-P subjects exhibited higher R2* values in the intermediate and posterior substantia nigra, whereas MSA-C subjects showed higher values in the ventral substantia nigra (Fig. 6a). No significant differences were found in the substantia nigra R2* values between iRBD and HCs.

## Discussion

Utilizing 7T T2*-weighted MRI, the present prospective diagnostic study assessed the diagnostic performance of DNH abnormality and explored the nigral iron deposition patterns in two independent cohorts, comprising 402 participants with PD, iRBD, and Parkinson-plus syndromes, as well as 100 control participants. This study represents the largest and the most comprehensive investigation of DNH to date. On 7T T2*-weighted MRI, we observed bilateral detectable DNH in 20.4% of early-stage PD, 79.5% of MSA-C, 93.5% of iRBD, and all HC participants. The 6-point visibility scale demonstrated an excellent diagnostic accuracy of 100% in distinguishing early-stage PD from HCs in both the development and the validation cohorts. This scale exhibited moderate performance in distinguishing between early-stage PD and iRBD or MSA-C, as well as between MSA-P or PSP and MSA-C. PD and PSP exhibited predominant iron deposition in the dorsal and posterior substantia nigra, while MSA-C showed predominant iron deposition in the ventral substantia nigra, with both the intermediate and posterior regions implicated in MSA-P.

We observed a relatively high DNH detectable rate in early-stage PD, with bilateral detection rate at 20.4% and unilateral detection rate at 34.3%, compared to previous 7T studies reporting bilateral detection in up to 6% [14] and unilateral detection in up to 17% [9] of early-stage PD cases. Several reasons could explain these differences, including our larger sample size and the use of 3D imaging rather than the 2D imaging employed in most prior studies [2, 6, 15]. The higher spatial resolution and signal-to-noise ratio, and the absence of crosstalk between sections in the 3D technique [30], enable a more accurate delineation of the DNH. The 6-point visibility scale achieved an accuracy of 100% in both development and validation cohorts for differentiating early-stage PD from HCs. Collectively, DNH impairment in early-stage PD appears to be primarily characterized by the degree of its impairment rather than its presence or absence. A previous study reported a positive correlation between 7T T2* signal change in nigrosome 1 and the total UPDRS scores in PD [6], suggesting that iron deposition in the substantia nigra of PD is a progressive process.

The DNH was bilaterally detected in 93.5% of iRBD patients in our study, with significantly lower rates of bilaterally and unilaterally normal DNH and 2-point area of hyperintensity scale score compared with HCs. These findings suggest that, despite an overall higher DNH detection rate in iRBD, there is considerable heterogeneity in the degree of DNH abnormality among iRBD patients. Previous studies have reported bilateral DNH detection rates ranging from 40% (6/15) [31] to 55% (16/29) [9] on 7T, and from 23.1% (3/13) [32] to 72.5% (29/40) [33] on 3 T in iRBD. The higher spatial resolution of 3D T2*-weighted sequence and patient heterogeneity may contribute to the high detection rate observed in our study. iRBD is a strong prodromal marker for α-synucleinopathies such as PD and MSA [34]. A prospective study of 1280 iRBD patients reported a conversion rate of 73.5% from iRBD to a neurodegenerative disease over a 12-year follow-up period [35]. The heterogeneity in DNH among iRBD patients suggests variations in nigral iron deposition, potentially linked to differences in the progression toward neurodegenerative disease. A susceptibility-weighted imaging and ^123^I-FP-CIT SPECT study revealed significantly lower putamenal dopaminergic activity in iRBD lacking DNH than those presenting with DNH [33]. Another study observed a loss of DNH in 89% (8/9) of iRBD with ^123^I-FP-CIT SPECT abnormality [31]. Thus, the DNH on 7T iron-sensitive MRI could be an alternative to DAT-PET/SPECT as an imaging marker, enabling direct visualization of prodromal dopaminergic deficits and prediction of conversion from iRBD to neurodegenerative diseases. During our follow-up evaluations in the iRBD group, two patients were diagnosed with PD (Table S11). One had bilateral absence of DNH on the baseline MRI and subsequently developed PD 10 months later. The other initially exhibited reduced DNH visibility, and converted to PD 14 months later. Conversion to parkinsonism (bradykinesia plus at least one of rest tremor or rigidity [20]) was not detected in other iRBD patients within two years of follow-up. Consistent with previous reports [35, 36], our cohort showed a similar conversion rate. These preliminary observations highlight the need for long-term, large-scale prospective cohort studies employing 7T MRI to further validate the prognostic utility of the DNH in predicting conversion from iRBD to overt parkinsonism.

Predominant loss of the DNH was found in MSA-P and PSP, and no significant variations in DNH abnormality were observed among PD, MSA-P, and PSP. Thus, the use of DNH alone may not be sufficient to distinguish between PD and MSA-P or PSP. This finding on 7T MRI aligns with previous 3 T studies, which reported limited discriminatory performance of the DNH abnormality in distinguishing between PD and Parkinson-plus syndrome [37–39]. In MSA-P, patients with bilateral detectable DNH had shorter disease duration, suggesting DNH preservation in early stages. In contrast, there were no significant differences in age at onset or disease duration between PSP patients with and without bilateral detectable DNH, indicating potential DNH heterogeneity among a subset of PSP cases at similar disease stages. Future longitudinal studies and subtype analyses are warranted to confirm these findings.

We also observed a high preservation rate of the DNH in MSA-C (79.5% bilaterally detected and 86.4% unilaterally detected). The DNH abnormality demonstrated moderate performance in distinguishing PD, MSA-P, and PSP from MSA-C in the pooled dataset. Pilot 7T studies have reported DNH detection rates ranging from 0% (0/1) to 20% (1/5) in MSA-C and 0% (0/2 and 0/5) in MSA-P [15, 16]. Nigrostriatal neuropathology is correlated with MSA-P, while the olivopontocerebellar system is more often affected in MSA-C [40]. A DAT SPECT study reported higher presynaptic nigrostriatal radiotracer uptake in MSA-C than PD and MSA-P [41]. Combining with the evidence that the loss of DNH is linked to nigrostriatal dopaminergic degeneration [42], the higher preservation rate of DNH in MSA-C may be explained by milder dopaminergic neuron degeneration and distinct nigral iron deposition patterns in comparison to MSA-P. Nevertheless, the heterogeneity of DNH abnormality in MSA-C may suggest the potential need for further subtyping in MSA-C. Further studies should investigate the differences in the pathophysiological mechanisms between MSA-C with and without DNH impairment, as well as the longitudinal alteration of this structure with disease progression. Overall, the preservation of DNH can serve as an imaging biomarker for classifying the two MSA subtypes.

A previous histological and postmortem 7T study identified DNH as nigrosome 1 [2], characterized by iron overload in PD [4]. The DNH abnormality on iron-sensitive MRI may serve as an imaging biomarker for dopaminergic degeneration [4]. In contrast, another study reported a partial overlap between DNH and nigrosome 1 following 3D reconstruction, with nigrosome 1 extending beyond DNH in both anteroposterior and superoinferior directions [3]. Although the anatomic structure corresponding to the DNH requires further investigation, DNH is a promising imaging biomarker for PD diagnosis.

PCA revealed distinctive nigral iron deposition patterns in PD, MSA-P, MSA-C, and PSP compared with HCs. Given that the DNH is located in the dorsal substantia nigra, increased iron deposition in the dorsal substantia nigra of PD, MSA-P, and PSP may explain the absence of the DNH in these conditions. A recent 7T study showed similar findings in PD, with pronounced neuromelanin signal loss in the lateral, posterior, and inferior part of SNpc, reflecting dopaminergic cell loss in these regions [43]. However, a postmortem study detected increased iron levels throughout the substantia nigra, rather than solely in the SNpc, in MSA [17]. Similarly, a histological study observed excessive iron deposition in the anterior substantia nigra of PSP [18]. These pathological studies, however, tended to include patients at advanced disease stages with longer disease durations and were often constrained by small sample sizes. Subregional differences in iron deposition in PD, iRBD, and Parkinson-plus syndromes, which may reflect distinct spatial development of substantia nigra pathology, warrant further investigation using 7T MRI, particularly in early-stage of these diseases.

Some limitations should be noted in this study. First, the diagnostic performance of the DNH and the patterns of nigral iron deposition need to be examined in long-term prospective cohort studies, especially in iRBD and early-stage PD. Second, the prognostic utility of the DNH in predicting conversion from iRBD to overt parkinsonism requires further long-term validation in larger, multi-center cohorts. Third, future investigations of the DNH abnormality should include corticobasal syndrome, dementia with Lewy bodies, and other diseases with striatal presynaptic dopaminergic dysfunction, such as hereditary parkinsonism [44], Wilson’s disease [45], and neurodegeneration with brain iron accumulation [46]. Furthermore, as 7T MRI is much more sensitive to motion artifacts, exclusion of subjects with severe cognitive impairment, tremor, or psychiatric disorders due to involuntary head movement could lead to selection bias. Lastly, microvessels have been recognized as a sporadic confounder in the assessment of the DNH [47], potentially affecting DNH assessment and R2* mapping.

## Conclusions

This large 7T MRI study reveals a relatively high detectable rate of the DNH in early-stage PD and its preservation in majority of MSA-C patients. The 6-point visibility scale holds potential as the optimal diagnostic method for early-stage PD. Impairment of the DNH in iRBD may indicate a higher risk of conversion to overt parkinsonism, although this observation requires confirmation through long-term follow-up studies. The DNH may remain detectable in the early stage of MSA-P, with potential heterogeneity observed in PSP. The patterns of nigral iron deposition for disease diagnosis and differential diagnosis require in-depth examination, considering the differences among PD, MSA-P, and MSA-C, as well as the overlapping between PD and PSP. Furthermore, as iron deposition in PD and Parkinson-plus syndromes is not limited to the substantia nigra and also involves other nuclei in the cerebral, brainstem, and cerebellum [48], it is necessary to investigate their potential as imaging biomarkers on 7T.

## Supplementary Information

Additional file 1. **eMethods.** **Table S1** Demographic and clinical characteristics in the pooled dataset. **Table S2** Inter-observer reliability of the seven DNH assessment methods in the pooled dataset. **Table S3** Visual assessment of the DNH in the development cohort at echo 1-echo 4. **Table S4** Visual assessment of the DNH in the pooled dataset at echo 1-echo 4. **Table S5** Visual assessment of the DNH in early-stage PD subtypes in the pooled dataset at echo 2. **Table S6** Predominant side of DNH impairment and motor symptoms. **Table S7** Diagnostic and differential diagnostic performances of the seven DNH assessment methods in the development cohort. **Table S8** Diagnostic and differential diagnostic performances of the seven DNH assessment methods in the pooled dataset. **Table S9** Validation of diagnostic and differential diagnostic performances of the DNH abnormality. **Table S10** Subgroup analyses of diagnostic and differential diagnostic performances of the seven DNH assessment methods in age- and education-matched participants aged over 60. **Table S11** Follow-up evaluations of iRBD patients. **Fig. S1** Flowchart of participant inclusion. **Fig. S2** 3D gradient-echo T2* images (echo 1-echo 4) for a representative case from each patient and healthy control groups. **Fig. S3** ROC curves for the three DNH rating scales at echo 2 in the pooled dataset. **Fig. S4** Receiver operating characteristic curves for the optimal DNH rating scale in the development cohort, its performance in the validation cohort, and its reassessment in the pooled dataset. **Analysis S1** Comparison of participant characteristics. **Analysis S2** Comparison of diagnostic and differential diagnostic performance between T2* echoes. **Analysis S3** Correlation analysis between clinical characteristics and DNH scores in PD and MSA-C patients. **Analysis S4** Comparison of clinical characteristics in MSA-P and PSP patients regarding the detectability of the DNH.

## Data Availability

The data that support the findings in this study are available from the corresponding author (TF) upon reasonable request.

## Abbreviations

AIC: Akaike information criterion
AUC: Area under the ROC curve
DNH: Dorsal nigral hyperintensity
HC: Healthy control
H-Y: Hoehn and Yahr
iRBD: Idiopathic rapid eye movement sleep behavior disorder
L R+: Positive likelihood ratio
LR-: Negative likelihood ratio
MDS-UPDRS: Movement disorder society-unified Parkinson’s disease rating scale
MSA: Multiple system atrophy
MSA-C: MSA-cerebellar type
MSA-P: MSA-parkinsonian type
PC: Principal component
PD: Parkinson’s disease
PSP: Progressive supranuclear palsy
ROC: Receiver operating characteristic
