# Supplementary material for: Dorsal hyperintensity and iron deposition patterns in the substantia nigra of Parkinson’s disease, idiopathic REM sleep behavior disorder, and Parkinson-plus syndromes at 7T MRI: a prospective diagnostic study
Source: Transl Neurodegener. 2025 Jul 4;14:35. doi: 10.1186/s40035-025-00495-4 (PMC12231697; doi:10.1186/s40035-025-00495-4)
Supplement: Supplementary file 1 — Additional file 1. eMethods. Table S1 Demographic and clinical characteristics in the pooled dataset. Table S2 Inter-observer reliability of the seven DNH assessment methods in the pooled dataset. Table S3 Visual assessment of the DNH in the development cohort at echo 1-echo 4. Table S4 Visual assessment of the DNH in the pooled dataset at echo 1-echo 4. Table S5 Visual assessment of the DNH in early-stage PD subtypes in the pooled dataset at echo 2. Table S6 Predominant side of DNH impairment and motor symptoms. Table S7 Diagnostic and differential diagnostic performances of the seven DNH assessment methods in the development cohort. Table S8 Diagnostic and differential diagnostic performances of the seven DNH assessment methods in the pooled dataset. Table S9 Validation of diagnostic and differential diagnostic performances of the DNH abnormality. Table S10 Subgroup analyses of diagnostic and differential diagnostic performances of the seven DNH assessment methods in age- and education-matched participants aged over 60. Table S11 Follow-up evaluations of iRBD patients. Fig. S1 Flowchart of participant inclusion. Fig. S2 3D gradient-echo T2* images (echo 1-echo 4) for a representative case from each patient and healthy control groups. Fig. S3 ROC curves for the three DNH rating scales at echo 2 in the pooled dataset. Fig. S4 Receiver operating characteristic curves for the optimal DNH rating scale in the development cohort, its performance in the validation cohort, and its reassessment in the pooled dataset. Analysis S1 Comparison of participant characteristics. Analysis S2 Comparison of diagnostic and differential diagnostic performance between T2* echoes. Analysis S3 Correlation analysis between clinical characteristics and DNH scores in PD and MSA-C patients. Analysis S4 Comparison of clinical characteristics in MSA-P and PSP patients regarding the detectability of the DNH. [file 40035_2025_495_MOESM1_ESM.docx]

**eMethods**

**MRI data acquisition**

Scans were performed on a 7T MRI scanner (MAGNETOM Terra, Siemens Healthineers, Germany). A 32-channel receive array head coil (Nova Medical, Wilmington, MA, USA) was used for signal acquisition. T1-weighted anatomic images were obtained using a three-dimensional (3D) magnetization-prepared rapid acquisition with gradient echo (MPRAGE) sequence, and the parameters were: echo time (TE) = 2.98 ms, repetition time (TR) = 2200 ms, inversion time (TI) = 1050 ms, flip angle (FA) = 6°, spatial resolution = 0.7 × 0.7 × 0.7 mm^3^, 256 sagittal slices, bandwidth = 260 Hz/pixel, and acquisition time = 6 minutes 12 seconds. All patients additionally underwent T1- and T2-weighted imaging on a 3T MRI scanner (MAGNETOM Prisma, Siemens Healthcare, Erlangen, Germany) using a 64-channel head/neck coil. The T1-weighted images were acquired using a 3D sagittal MPRAGE sequence with the following parameters: TE/TR/TI = 2.0/2000/880 ms, flip angle = 8°, voxel size (AP×RL×SI) = 1×1×1 mm^3^, FOV (AP×RL×SI) = 240×208×256 mm^3^, and acceleration factor = 2. The 2D T2-weighted images were acquired using axial motion-insensitive turbo spin-echo sequence (BLADE) (1) with the following parameters: TE/TR = 111/4000 ms, flip angle = 90°, voxel size (AP×RL×SI) = 0.6×0.6×4.0 mm3, FOV (AP×RL) = 220×220 mm2, slice number = 36, gap = 0, and acceleration factor = 3.

**Visual Assessment of the DNH**

Abnormality of the dorsal nigral hyperintensity (DNH) was assessed based on the following seven methods:

1. The DNH was rated dichotomously as “non-pathological” or “pathological” according to whether its hyperintensity could be visually detected (ie, presence of its hyperintense signal even if it may exhibit blurred outline or reduced size within its presumed anatomical region) (2,3). We further classified this method into two subcategories:
2. bilaterally detected: non-pathological = the DNH could be visually detected on both sides of the substantia nigra, pathological = the DNH could not be visually detected on one side or both sides of the substantia nigra;

2) unilaterally detected: non-pathological = the DNH could be visually detected on one side or both sides of the substantia nigra, pathological = the DNH could not be visually detected on both sides of the substantia nigra.

(2) The DNH was rated dichotomously as “non-pathological” or “pathological” according to whether the hyperintensity signal maintains its ovoid structure, appears normally bright with a clear outline, and shows no size reduction within its presumed anatomical region (4). This method was also classified into two subcategories:

1) bilaterally normal: non-pathological = the DNH was normally bright and present on both sides of the substantia nigra, pathological = the DNH was not normally bright and was absent on one side or both sides of the substantia nigra.

2) unilaterally normal: non-pathological = the DNH was normally bright and present on one side or both sides of the substantia nigra, pathological = the DNH was not normally bright and was absent on both sides of the substantia nigra.

(3) Evaluation by a 6-point visibility scale (4) for each hemi-mesencephalon: 5 = normally bright and present, 4 = reduced size or slightly more difficult to see than normal but definitely identifiable, 3 = very difficult to detect but identifiable, 2 = parts of the outline possibly visible but not definitely identifiable, 1 = not identifiable from the surrounding low signal, 0 = darker than the surrounding substantia nigra.

(4) Evaluation by the area of hyperintensity using a 2-point scale (5) for each hemi-mesencephalon: 2 = normally bright and present, 1 = hypointensity in < 50% of the presumed DNH region, 0 = hypointensity in ≥ 50% of the presumed DNH region.

(5) Evaluation by a 2-point swallow tail sign scale (6–8) for each hemi-mesencephalon: 2 = normally bright and present, 1 = blurred outline and decreased hyperintensity but still identifiable, 0 = absence of the hyperintense signal.

**Image Postprocessing**

**Construction of R2* mapping**

Firstly, T1 anatomic imaging was registered to the MNI template using non-linear registration and the registration parameters were saved. The first echo (echo 1) of the multi-echo T2* images were then registered to T1 anatomic images using rigid-body registration and the registration parameters were saved. Then, these two sets of registration parameters were combined to transform the multi-echo T2* images to MNI space with 0.5mm resolution in one step. All registration procedures were done using the ANTs toolbox (9) (available at https://github.com/ANTsX/ANTs). Then, the R2* map was calculated using the 4-echo T2*-weighted images in MNI space using a linear regression model with the SEPIA toolbox (10) (available at: https://github.com/kschan0214/sepia).

**Principal component analysis of the substantia nigra**

To investigate the alterations of iron deposition patterns in PD, idiopathic rapid eye movement sleep behavior disorder (iRBD), and Parkinson-plus syndromes, we performed voxel-wise principal component analysis (PCA) on 0.5mm MNI-space nigral R2* map. The comparisons were made between various disease groups (early-stage PD, advanced PD, iRBD, multiple system atrophy-parkinsonian type [MSA-P], multiple system atrophy-cerebellar type [MSA-C], and progressive supranuclear palsy [PSP]) and the healthy control (HC) group. Based on the “atlasing of the basal ganglia (ATAG)” data set (11) (available at: https://www.nitrc.org/projects/atag/), a mask of the substantia nigra was generated using MIAKAT (www.miakat.org) to isolate voxels and to acquire the 3D voxel-wise images from the 0.5mm MNI-space R2* map. Images were then smoothed using a 3D Gaussian filter with a sigma level (kernel size) of 0.5. To effectively capture the differences in R2* among different diseases, principal components (PCs) were identified based on the Akaike information criterion (AIC) model, a mathematical model that evaluates how well a particular model fits the data and compares its quality to other models derived from the same dataset (12). In this study, the AIC was utilized to determine the optimal combination of PCs that explained the most significant variance in the data. It compared PCs with each other to identify the most relevant combination. To narrow down the selection of PCs, an additional threshold was set. Only PCs that showed a significance level below *p* = 0.2 in separating patients from HC and accounted for at least 5% of the data variance were evaluated by the AIC model. Each selected PC combination was further validated by conducting an independent samples t-test on subject scores to ensure a statistically significant separation (*P* < 0.05) between the two subject cohorts. The resulting 3D voxel-wise images displayed the spatial distribution of voxels with the combined PC weightings, which effectively allowed the differentiation between the two cohorts after applying a similar z-score transformation. To validate the performance of the PCA, jackknife resampling was employed. This technique estimates the variance of statistics derived from the entire dataset, providing additional confidence in the reliability of the PCA results (13). The jackknife resampling technique involves systematically leaving out one observation from the dataset at a time and re-estimating the mean and variance of the PCA regional and voxel-wise weights. This process is repeated n times, where n represents the total number of observations in the dataset (13). A one-sample t-test was conducted across all n-1 voxel-wise PCA iterations to assess the reliability of the calculated PCA weights. The α level was set to 0.0001 to minimize the false-positive rate (14).

**Table S1** Demographic and clinical characteristics in the pooled dataset

|  | **PD** | | | **iRBD** | **MSA-P** | **MSA-C** | **PSP** | **HC** |
| --- | --- | --- | --- | --- | --- | --- | --- | --- |
|  | **Total** | **Early-stage PD** | **Advanced PD** |  |  |  |  |  |
|  | ***n*=162** | ***n =* 108** | ***n =* 54** | ***n =* 31** | ***n =* 87** | ***n =* 88** | ***n =* 34** | ***n =* 100** |
| Age, year, median (IQR) | 60.5 (54.0-66.0) | 59.0 (52.0-64.8) | 65.0 (59.0-68.3) | 64.0 (58.0-68.0) | 59.0 (54.0-66.0) | 58.0 (53.0-64.8) | 68.5 (62.8-72.0) | 60.0 (54.3-66.0) |
| Sex, male/female | 84/78 | 55/53 | 29/25 | 20/11 | 35/52 | 45/43 | 22/12 | 41/59 |
| Education, year, median (IQR) | 12.0 (9.0-15.3) | 12.0 (9.0-15.3) | 12.0 (9.0-15.8) | 12.0 (9.0-15.8) | 12.0 (9.0-12.0) | 9.5 (9.0-12.0) | 9.0 (7.5-12.0) | 12.0 (9.0-16.0) |
| Disease duration, year, median (IQR) | 3.0 (1.8-6.0) | 2.0 (1.0-3.0) | 8.0 (6.0-10.0) | 3.0 (1.0-5.0) | 2.0 (1.5-3.0) | 2.0 (1.0-3.0) | 2.0 (2.0-4.3) | - |
| MDS-UPDRS III (OFF), median (IQR) | 28 (19-39) | 24 (15-31) | 40 (32-53) | 2 (1-5) | 44 (34-57) | 25 (18-31) | 38 (29-51) | - |
| H-Y stage (OFF), median (IQR) | 2.0 (2.0-2.5) | 2.0 (2.0-2.0) | 3.0 (2.5-3.0) | - | - | - | - | - |
| MMSE, median (IQR) | 28 (26-29) | 29 (26-30) | 28 (26-29) | 29 (27-29) | 28 (25-29) | 28 (26-29) | 24 (22-27) | 29 (28-29) |
| MoCA, median (IQR) | 23 (20-26) | 24 (20-27) | 22 (19-26) | 25 (22-27) | 23 (20-26) | 22 (19-25) | 19 (15-22) | 25 (22-27) |
| HAMA, median (IQR) | 6 (4-10) | 6 (4-10) | 7 (4-12) | 8 (3-11) | 8 (5-11) | 7 (4-11) | 6 (4-11) | 4 (1-6) |
| HAMD, median (IQR) | 6 (3-11) | 6 (3-11) | 6 (3-9) | 7 (3-10) | 8 (5-13) | 7 (4-12) | 8 (5-12) | 3 (1-7) |

PD, Parkinson’s disease; iRBD, idiopathic rapid eye movement sleep behavior disorder; MSA, multiple system atrophy; MSA-P, MSA-parkinsonian type; MSA-C, MSA-cerebellar type; PSP, progressive supranuclear palsy; HC, healthy control; MDS-UPDRS, Movement Disorder Society-Unified Parkinson’s Disease Rating Scale; H-Y, Hoehn and Yahr; MMSE, Mini-Mental State Examination; MoCA, Montreal Cognitive Assessment; HAMD, Hamilton Depression Rating Scale; HAMA, Hamilton Anxiety Rating Scale; n, number; IQR, interquartile range.

**Table S2** Inter-observer reliability of the seven DNH assessment methods in the pooled dataset

| Methods | Echo 1 | Echo 2 | Echo 3 | Echo 4 |
| --- | --- | --- | --- | --- |
| Bilaterally detected ^a^ | 0.984 | 0.984 | 0.992 | 0.987 |
| Unilaterally detected ^a^ | 0.980 | 0.980 | 0.984 | 0.984 |
| Bilaterally normal ^a^ | 0.913 | 0.946 | 0.949 | 0.924 |
| Unilaterally normal ^a^ | 0.879 | 0.953 | 0.935 | 0.935 |
| 6-point visibility scale ^b^ | 0.960 | 0.977 | 0.975 | 0.980 |
| 2-point area of hyperintensity scale ^b^ | 0.940 | 0.972 | 0.960 | 0.963 |
| 2-point swallow tail sign scale ^b^ | 0.956 | 0.973 | 0.975 | 0.976 |

^a^ Cohen’s kappa coefficient.

^b^ Weighted Cohen’s kappa coefficient.

DNH, dorsal nigral hyperintensity.

**Table S3** Visual assessment of the DNH in the development cohort at echo 1-echo 4

|  | **Early-stage PD** | **Advanced PD** | **iRBD** | **MSA-P** | **MSA-C** | **PSP** | **HC** |
| --- | --- | --- | --- | --- | --- | --- | --- |
|  | ***n =* 62** | ***n =* 54** | ***n =* 31** | ***n =* 53** | ***n =* 52** | ***n =* 34** | ***n =* 60** |
| Bilaterally detected, n (%) |  |  |  |  |  |  |  |
| Echo 1 | 14 (22.6) ^a, b, c^ | 3 (5.6) ^a, b, c^ | 29 (93.5) | 3 (5.7) ^a, b, c^ | 41 (78.8) ^a^ | 4 (11.8) ^a, b, c^ | 60 (100) |
| Echo 2 | 14 (22.6) ^a, b, c^ | 2 (3.7) ^a, b, c^ | 29 (93.5) | 3 (5.7) ^a, b, c^ | 41 (78.8) ^a^ | 4 (11.8) ^a, b, c^ | 60 (100) |
| Echo 3 | 10 (16.1) ^a, b, c^ | 0 (0) ^a, b, c, d^ | 29 (93.5) | 1 (1.9) ^a, b, c^ | 37 (71.2) ^a^ | 2 (5.9) ^a, b, c^ | 60 (100) |
| Echo 4 | 4 (6.5) ^a, b, c^ | 0 (0) ^a, b, c^ | 26 (83.9) | 0 (0) ^a, b, c^ | 25 (48.1) ^a, b^ | 2 (5.9) ^a, b, c^ | 59 (98.3) |
| Unilaterally detected, n (%) |  |  |  |  |  |  |  |
| Echo 1 | 23 (37.1) ^a, b, c^ | 7 (13.0) ^a, b, c^ | 29 (93.5) | 9 (17.0) ^a, b, c^ | 45 (86.5) | 5 (14.7) ^a, b, c^ | 60 (100) |
| Echo 2 | 23 (37.1) ^a, b, c^ | 7 (13.0) ^a, b, c^ | 29 (93.5) | 9 (17.0) ^a, b, c^ | 45 (86.5) | 5 (14.7) ^a, b, c^ | 60 (100) |
| Echo 3 | 20 (32.3) ^a, b, c^ | 4 (7.4) ^a, b, c, d^ | 29 (93.5) | 8 (15.1) ^a, b, c^ | 45 (86.5) | 4 (11.8) ^a, b, c^ | 60 (100) |
| Echo 4 | 12 (19.4) ^a, b, c^ | 1 (1.9) ^a, b, c^ | 27 (87.1) | 4 (7.5) ^a, b, c^ | 35 (67.3) ^a^ | 3 (8.8) ^a, b, c^ | 60 (100) |
| Bilaterally normal, n (%) |  |  |  |  |  |  |  |
| Echo 1 | 0 (0) ^a^ | 0 (0) ^a^ | 3 (9.7) ^a^ | 0 (0) ^a^ | 1 (1.9) ^a^ | 1 (2.9) ^a^ | 37 (61.7) |
| Echo 2 | 0 (0) ^a, b^ | 0 (0) ^a, b^ | 5 (16.1) ^a^ | 0 (0) ^a^ | 4 (7.7) ^a^ | 1 (2.9) ^a^ | 41 (68.3) |
| Echo 3 | 0 (0) ^a^ | 0 (0) ^a^ | 2 (6.5) ^a^ | 0 (0) ^a^ | 2 (3.8) ^a^ | 0 (0) ^a^ | 27 (45.0) |
| Echo 4 | 0 (0) ^a^ | 0 (0) ^a^ | 1 (3.2) | 0 (0) ^a^ | 1 (1.9) | 0 (0) | 10 (16.7) |
| Unilaterally normal, n (%) |  |  |  |  |  |  |  |
| Echo 1 | 0 (0) ^a, b^ | 0 (0) ^a, b^ | 5 (16.1) ^a^ | 0 (0) ^a^ | 4 (7.7) ^a^ | 1 (2.9) ^a^ | 44 (73.3) |
| Echo 2 | 0 (0) ^a, b, c^ | 0 (0) ^a, b, c^ | 8 (25.8) ^a^ | 0 (0) ^a, b, c^ | 10 (19.2) ^a^ | 1 (2.9) ^a^ | 50 (83.3) |
| Echo 3 | 0 (0) ^a^ | 0 (0) ^a^ | 2 (6.5) ^a^ | 0 (0) ^a^ | 3 (5.8) ^a^ | 0 (0) ^a^ | 35 (58.3) |
| Echo 4 | 0 (0) ^a^ | 0 (0) ^a^ | 2 (6.5) | 0 (0) ^a^ | 1 (1.9) ^a^ | 0 (0) ^a^ | 17 (28.3) |
| 6-point visibility scale, median (IQR) |  |  |  |  |  |  |  |
| Echo 1 | 2 (2-4) ^a, b, c^ | 2 (2-2) ^a, b, c^ | 7 (6-8) | 2 (2-2) ^a, b, c^ | 6 (4-8) ^a^ | 2 (2-2) ^a, b, c^ | 10 (8-10) |
| Echo 2 | 2 (2-4) ^a, b, c^ | 2 (2-2) ^a, b, c^ | 7 (6-9) | 2 (2-2) ^a, b, c^ | 7 (5-8) ^a^ | 2 (2-2) ^a, b, c^ | 10 (9-10) |
| Echo 3 | 2 (2-3) ^a, b, c^ | 2 (2-2) ^a, b, c^ | 6 (5-8) | 2 (2-2) ^a, b, c^ | 5 (4-7) ^a^ | 2 (2-2) ^a, b, c^ | 9 (8-10) |
| Echo 4 | 2 (2-2) ^a, b, c^ | 2 (2-2) ^a, b, c^ | 5 (4-6) | 2 (2-2) ^a, b, c^ | 3 (2-6) ^a^ | 2 (2-2) ^a, b, c^ | 8 (6-9) |
| 2-point area of hyperintensity scale, median (IQR) |  |  |  |  |  |  |  |
| Echo 1 | 0 (0-0) ^a, b^ | 0 (0-0) ^a, b^ | 1 (0-2) ^a^ | 0 (0-0) ^a, b^ | 0 (0-2) ^a^ | 0 (0-0) ^a, b^ | 4 (2-4) |
| Echo 2 | 0 (0-0) ^a, b, c^ | 0 (0-0) ^a, b, c^ | 1 (0-3) ^a^ | 0 (0-0) ^a, b, c^ | 1 (0-2) ^a^ | 0 (0-0) ^a, b, c^ | 4 (3-4) |
| Echo 3 | 0 (0-0) ^a, b^ | 0 (0-0) ^a, b^ | 0 (0-2) ^a^ | 0 (0-0) ^a, b^ | 0 (0-1) ^a^ | 0 (0-0) ^a, b^ | 3 (2-4) |
| Echo 4 | 0 (0-0) ^a^ | 0 (0-0) ^a^ | 0 (0-0) ^a^ | 0 (0-0) ^a^ | 0 (0-0) ^a^ | 0 (0-0) ^a^ | 2 (0-3) |
| 2-point swallow tail sign scale, median (IQR) |  |  |  |  |  |  |  |
| Echo 1 | 0 (0-1) ^a, b, c^ | 0 (0-0) ^a, b, c^ | 2 (2-2) | 0 (0-0) ^a, b, c^ | 2 (2-2) ^a^ | 0 (0-0) ^a, b, c^ | 4 (2-4) |
| Echo 2 | 0 (0-1) ^a, b, c^ | 0 (0-0) ^a, b, c^ | 2 (2-3) | 0 (0-0) ^a, b, c^ | 2 (2-2) ^a^ | 0 (0-0) ^a, b, c^ | 4 (3-4) |
| Echo 3 | 0 (0-1) ^a, b, c^ | 0 (0-0) ^a, b, c^ | 2 (2-2) | 0 (0-0) ^a, b, c^ | 2 (1-2) ^a^ | 0 (0-0) ^a, b, c^ | 3 (2-4) |
| Echo 4 | 0 (0-0) ^a, b, c^ | 0 (0-0) ^a, b, c^ | 2 (2-2) | 0 (0-0) ^a, b, c^ | 1 (0-2) ^a^ | 0 (0-0) ^a, b, c^ | 2 (2-3) |

^a^ *P* < 0.05 compared with HC.

^b^ *P* < 0.05 compared with iRBD.

^c^ *P* < 0.05 compared with MSA-C.

^d^ *P* < 0.05 compared with early-stage PD.

Chi-squared test or Fisher’s exact test, with Bonferroni correction applied for multiple comparisons, was used to compare dichotomous evaluation methods. Kruskal-Wallis test with Bonferroni correction was used to compare DNH rating scale scores.

DNH, dorsal nigral hyperintensity; PD, Parkinson’s disease; iRBD, idiopathic rapid eye movement sleep behavior disorder; MSA, multiple system atrophy; MSA-P, MSA-parkinsonian type; MSA-C, MSA-cerebellar type; PSP, progressive supranuclear palsy; HC, healthy control; n, number; IQR, interquartile range.

**Table S4** Visual assessment of the DNH in the pooled dataset at echo 1-echo 4

|  | **PD** | | | **iRBD** | **MSA-P** | **MSA-C** | **PSP** | **HC** |
| --- | --- | --- | --- | --- | --- | --- | --- | --- |
|  | **Total** | **Early-stage PD** | **Advanced PD** |  |  |  |  |  |
|  | ***n*=162** | ***n =* 108** | ***n =* 54** | ***n =* 31** | ***n =* 87** | ***n =* 88** | ***n =* 34** | ***n =* 100** |
| Bilaterally detected, n (%) |  |  |  |  |  |  |  |  |
| Echo 1 | 25 (15.4) a | 22 (20.4) a, b, c | 3 (5.6) a, b, c | 29 (93.5) | 6 (6.9) a, b, c | 70 (79.5) a | 4 (11.8) a, b, c | 100 (100) |
| Echo 2 | 24 (14.8) a | 22 (20.4) a, b, c | 2 (3.7) a, b, c | 29 (93.5) | 5 (5.7) a, b, c | 70 (79.5) a | 4 (11.8) a, b, c | 100 (100) |
| Echo 3 | 15 (9.3) a | 15 (13.9) a, b, c | 0 (0) a, b, c | 29 (93.5) | 2 (2.3) a, b, c | 61 (69.3) a | 2 (5.9) a, b, c | 100 (100) |
| Echo 4 | 6 (3.7) a | 6 (5.6) a, b, c | 0 (0) a, b, c | 26 (83.9) a | 1 (1.1) a, b, c | 48 (54.5) a | 2 (5.9) a, b, c | 99 (99.0) |
| Unilaterally detected, n (%) |  |  |  |  |  |  |  |  |
| Echo 1 | 44 (27.2) a | 37 (34.3) a, b, c | 7 (13.0) a, b, c | 29 (93.5) | 14 (16.1) a, b, c | 76 (86.4) a | 5 (14.7) a, b, c | 100 (100) |
| Echo 2 | 44 (27.2) a | 37 (34.3) a, b, c | 7 (13.0) a, b, c | 29 (93.5) | 14 (16.1) a, b, c | 76 (86.4) a | 5 (14.7) a, b, c | 100 (100) |
| Echo 3 | 34 (21.0) a | 30 (27.8) a, b, c | 4 (7.4) a, b, c | 29 (93.5) | 12 (13.8) a, b, c | 74 (84.1) a | 4 (11.8) a, b, c | 100 (100) |
| Echo 4 | 21 (13.0) a | 20 (18.5) a, b, c | 1 (1.9) a, b, c | 27 (87.1) a | 8 (9.2) a, b, c | 61 (69.3) a | 3 (8.8) a, b, c | 100 (100) |
| Bilaterally normal, n (%) |  |  |  |  |  |  |  |  |
| Echo 1 | 0 (0) a | 0 (0) a, b | 0 (0) a | 3 (9.7) a | 0 (0) a | 4 (4.5) a | 1 (2.9) a | 55 (55.0) |
| Echo 2 | 0 (0) a | 0 (0) a, b | 0 (0) a, b | 5 (16.1) a | 0 (0) a, b | 7 (8.0) a | 1 (2.9) a | 66 (66.0) |
| Echo 3 | 0 (0) a | 0 (0) a | 0 (0) a | 2 (6.5) a | 0 (0) a | 4 (4.5) a | 0 (0) a | 39 (39.0) |
| Echo 4 | 0 (0) a | 0 (0) a | 0 (0) a | 1 (3.2) a | 0 (0) a | 4 (4.5) | 0 (0) a | 17 (17.0) |
| Unilaterally normal, n (%) |  |  |  |  |  |  |  |  |
| Echo 1 | 0 (0) a | 0 (0) a, b, c | 0 (0) a | 5 (16.1) a | 0 (0) a, b, c | 10 (11.4) a | 1 (2.9) a | 73 (73.0) |
| Echo 2 | 0 (0) a | 0 (0) a, b, c | 0 (0) a, b, c | 8 (25.8) a | 0 (0) a, b, c | 18 (20.5) a | 1 (2.9) a | 83 (83.0) |
| Echo 3 | 0 (0) a | 0 (0) a | 0 (0) a | 2 (6.5) a | 0 (0) a | 6 (6.8) a | 0 (0) a | 55 (55.0) |
| Echo 4 | 0 (0) a | 0 (0) a | 0 (0) a | 2 (6.5) | 0 (0) a | 4 (4.5) a | 0 (0) a | 29 (29.0) |
| 6-point visibility scale, median (IQR) |  |  |  |  |  |  |  |  |
| Echo 1 | 2 (2-3) a | 2 (2-4) a, b, c | 2 (2-2) a, b, c | 7 (6-8) | 2 (2-2) a, b, c | 6 (4-8) a | 2 (2-2) a, b, c | 10 (8-10) |
| Echo 2 | 2 (2-3) a | 2 (2-4) a, b, c | 2 (2-2) a, b, c | 7 (6-9) a | 2 (2-2) a, b, c | 7 (5-8) a | 2 (2-2) a, b, c | 10 (9-10) |
| Echo 3 | 2 (2-2) a | 2 (2-3) a, b, c | 2 (2-2) a, b, c | 6 (5-8) | 2 (2-2) a, b, c | 5 (3-7) a | 2 (2-2) a, b, c | 9 (8-10) |
| Echo 4 | 2 (2-2) a | 2 (2-2) a, b, c | 2 (2-2) a, b, c | 5 (4-6) a | 2 (2-2) a, b, c | 4 (2-6) a | 2 (2-2) a, b, c | 8 (6-9) |
| 2-point area of hyperintensity scale, median (IQR) |  |  |  |  |  |  |  |  |
| Echo 1 | 0 (0-0) a | 0 (0-0) a, b, c | 0 (0-0) a, b, c | 1 (0-2) a | 0 (0-0) a, b, c | 0 (0-2) a | 0 (0-0) a, b, c | 4 (2-4) |
| Echo 2 | 0 (0-0) a | 0 (0-0) a, b, c | 0 (0-0) a, b, c | 1 (0-3) a | 0 (0-0) a, b, c | 1 (0-2) a | 0 (0-0) a, b, c | 4 (3-4) |
| Echo 3 | 0 (0-0) a | 0 (0-0) a, b, c | 0 (0-0) a, b, c | 0 (0-2) a | 0 (0-0) a, b, c | 0 (0-1) a | 0 (0-0) a, b, c | 3 (2-4) |
| Echo 4 | 0 (0-0) a | 0 (0-0) a | 0 (0-0) a | 0 (0-0) a | 0 (0-0) a | 0 (0-0) a | 0 (0-0) a | 2 (0-3) |
| 2-point swallow tail sign scale, median (IQR) |  |  |  |  |  |  |  |  |
| Echo 1 | 0 (0-1) a | 0 (0-1) a, b, c | 0 (0-0) a, b, c | 2 (2-2) | 0 (0-0) a, b, c | 2 (2-2) a | 0 (0-0) a, b, c | 4 (2-4) |
| Echo 2 | 0 (0-1) a | 0 (0-1) a, b, c | 0 (0-0) a, b, c | 2 (2-3) a | 0 (0-0) a, b, c | 2 (2-2) a | 0 (0-0) a, b, c | 4 (3-4) |
| Echo 3 | 0 (0-0) a | 0 (0-1) a, b, c | 0 (0-0) a, b, c | 2 (2-2) | 0 (0-0) a, b, c | 2 (1-2) a | 0 (0-0) a, b, c | 3 (2-4) |
| Echo 4 | 0 (0-0) a | 0 (0-0) a, b, c | 0 (0-0) a, b, c | 2 (2-2) | 0 (0-0) a, b, c | 2 (0-2) a | 0 (0-0) a, b, c | 2 (2-3) |

^a^ *P* < 0.05 compared with HC.

^b^ *P* < 0.05 compared with iRBD.

^c^ *P* < 0.05 compared with MSA-C.

Chi-squared test or Fisher’s exact test, with Bonferroni correction applied for multiple comparisons, was used to compare dichotomous evaluation methods. Kruskal-Wallis test with Bonferroni correction was used to compare DNH rating scale scores.

DNH, dorsal nigral hyperintensity; PD, Parkinson’s disease; iRBD, idiopathic rapid eye movement sleep behavior disorder; MSA, multiple system atrophy; MSA-P, MSA-parkinsonian type; MSA-C, MSA-cerebellar type; PSP, progressive supranuclear palsy; HC, healthy control; n, number; IQR, interquartile range.

**Table S5** Visual assessment of the DNH in early-stage PD subtypes in the pooled dataset at echo 2

| Methods | Tremor-dominant ^a^ early-stage PD | Non-tremor-dominant ^b^ early-stage PD | *p* |
| --- | --- | --- | --- |
|  | *n =* 43 | *n =* 65 |  |
| Bilaterally detected, n (%) | 10 (23.3) | 12 (18.5) | 0.545^c^ |
| Unilaterally detected, n (%) | 18 (41.9) | 19 (29.2) | 0.176^c^ |
| Bilaterally normal, n (%) | 0 (0) | 0 (0) | - |
| Unilaterally normal, n (%) | 0 (0) | 0 (0) | - |
| 6-point visibility scale, median (IQR) | 2 (2-4) | 2 (2-4) | 0.191^d^ |
| 2-point area of hyperintensity scale, median (IQR) | 0 (0-0) | 0 (0-0) | 0.539^d^ |
| 2-point swallow tail sign scale, median (IQR) | 0 (0-1) | 0 (0-1) | 0.222^d^ |

^a^ Ratio of the mean tremor scores to the mean PIGD scores ≥1.15 according to the MDS-UPDRS (15).

^b^ Ratio of the mean tremor scores to the mean PIGD scores <1.15 according to the MDS-UPDRS.

^c^ Chi-squared test.

^d^ Mann-Whitney U test.

DNH, dorsal nigral hyperintensity; PD, Parkinson’s disease; PIGD, postural instability and gait disturbance; MDS-UPDRS, Movement Disorders Society Unified Parkinson’s Disease Rating Scale; n, number; IQR, interquartile range.

**Table S6** Predominant side of DNH impairment and motor symptoms

|  | **PD** | **MSA-P** | **MSA-C** | **PSP** | **Total** |
| --- | --- | --- | --- | --- | --- |
|  | ***n =* 25** | ***n =* 12** | ***n =* 32** | ***n =* 2** | ***n =* 71** |
| Predominant side of DNH impairment according to 6-point visibility scale, left/right | 14/11 | 3/9 | 18/14 | 1/1 | 36/35 |
| Predominant side of motor symptoms * |  |  |  |  |  |
| Total, left/right | 9/16 | 8/2 | 5/2 | 1/1 | 23/21 |
| Rigidity, left/right | 7/14 | 7/2 | 3/0 | 1/1 | 18/17 |
| Bradykinesia, left/right | 9/16 | 8/1 | 6/2 | 1/1 | 24/20 |
| Tremor, left/right | 8/15 | 4/0 | 1/1 | 0/0 | 13/16 |

* Predominant side of motor symptoms was evaluated according to MDS-UPDRS III subscores, with rigidity evaluated by item 3.3; bradykinesia evaluated by items 3.4, 3.5, 3.6, 3.7, and 3.8; and tremor evaluated by items 3.15, 3.16, and 3.17.

DNH, dorsal nigral hyperintensity; PD, Parkinson’s disease; MSA, multiple system atrophy; MSA-P, MSA-parkinsonian type; MSA-C, MSA-cerebellar type; PSP, progressive supranuclear palsy; MDS-UPDRS, Movement Disorders Society Unified Parkinson’s Disease Rating Scale.

A total of 71 patients across disease groups with unilateral detectable DNH were evaluated for consistency between the predominant side of DNH impairment and the lateralization of motor symptoms. Aside from the 27 patients (2 MSA-P and 25 MSA-C) exhibiting bilaterally symmetric motor symptoms, 84.1% (37/44) patients demonstrated a contralateral relationship between the predominant side of DNH impairment and the predominant side of motor symptoms. In the PD group, the rate was 84.0% (21/25) for the overall motor symptoms, 81.0% (17/21) for rigidity, 84.0% (21/25) for bradykinesia, and 87.0% (20/23) for tremor.

**Table S7 Diagnostic and differential diagnostic performances of the seven DNH assessment methods in the development cohort**

|  | Methods | DNH score | Sensitivity (%) | Specificity (%) | LR+ | LR- | PPV (%) | NPV (%) | Accuracy (%) | AUC |
| --- | --- | --- | --- | --- | --- | --- | --- | --- | --- | --- |
| Early-stage PD vs. HC | bilaterally detected | - | 77.4 | 100 | >10 | 0.23 | 100 | 81.1 | 88.5 | - |
|  | unilaterally detected | - | 62.9 | 100 | >10 | 0.37 | 100 | 72.3 | 81.1 | - |
|  | bilaterally normal | - | 100 | 68.3 | 3.16 | 0 | 76.5 | 100 | 84.4 | - |
|  | unilaterally normal ^b^ | - | 100 | 83.3 | 6 | 0 | 86.1 | 100 | 91.8 | - |
|  | 6-point visibility scale ^a,*^ | 6 | 100 | 100 | >10 | 0 | 100 | 100 | 100 | 1.000 |
|  | 2-point area of hyperintensity scale ^a,*^ | 1 | 100 | 98.3 | 60 | 0 | 98.4 | 100 | 99.2 | 1.000 |
|  | 2-point swallow tail sign scale ^b^ | 2 | 100 | 83.3 | 6 | 0 | 86.1 | 100 | 91.8 | 0.981 |
| Advanced PD vs. HC | bilaterally detected ^a,*^ | - | 96.3 | 100 | >10 | 0.04 | 100 | 96.8 | 98.2 | - |
|  | unilaterally detected ^b,*^ | - | 87.0 | 100 | >10 | 0.13 | 100 | 89.6 | 93.9 | - |
|  | bilaterally normal | - | 100 | 68.3 | 3.16 | 0 | 74.0 | 100 | 83.3 | - |
|  | unilaterally normal ^b^ | - | 100 | 83.3 | 6 | 0 | 84.4 | 100 | 91.2 | - |
|  | 6-point visibility scale ^a,*^ | 5 | 100 | 100 | >10 | 0 | 100 | 100 | 100 | 1.000 |
|  | 2-point area of hyperintensity scale ^a,*^ | 1 | 100 | 98.3 | 60 | 0 | 98.2 | 100 | 99.1 | 1.000 |
|  | 2-point swallow tail sign scale ^a,*^ | 1 | 96.3 | 100 | >10 | 0.04 | 100 | 96.8 | 98.2 | 0.997 |
| MSA-P vs. HC | bilaterally detected ^a,*^ | - | 94.3 | 100 | >10 | 0.06 | 100 | 95.2 | 97.3 | - |
|  | unilaterally detected ^b^ | - | 83.0 | 100 | >10 | 0.17 | 100 | 87.0 | 92.0 | - |
|  | bilaterally normal | - | 100 | 68.3 | 3.16 | 0 | 73.6 | 100 | 83.2 | - |
|  | unilaterally normal ^b^ | - | 100 | 83.3 | 6 | 0 | 84.1 | 100 | 91.2 | - |
|  | 6-point visibility scale ^a,*^ | 6 | 100 | 100 | >10 | 0 | 100 | 100 | 100 | 1.000 |
|  | 2-point area of hyperintensity scale ^a,*^ | 1 | 100 | 98.3 | 60 | 0 | 98.1 | 100 | 99.1 | 1.000 |
|  | 2-point swallow tail sign scale ^a,*^ | 1 | 94.3 | 100 | >10 | 0.06 | 100 | 95.2 | 97.3 | 0.995 |
| MSA-C vs. HC | bilaterally detected | - | 21.2 | 100 | >10 | 0.79 | 100 | 59.4 | 63.4 | - |
|  | unilaterally detected | - | - | - | - | - | - | - | - | - |
|  | bilaterally normal | - | 92.3 | 68.3 | 2.91 | 0.11 | 71.6 | 91.1 | 79.5 | - |
|  | unilaterally normal | - | 80.8 | 83.3 | 4.85 | 0.23 | 80.8 | 83.3 | 82.1 | - |
|  | 6-point visibility scale | 8 | 80.8 | 81.7 | 4.41 | 0.24 | 79.2 | 83.1 | 81.3 | 0.897 |
|  | 2-point area of hyperintensity scale | 2 | 80.8 | 81.7 | 4.41 | 0.24 | 79.2 | 83.1 | 81.3 | 0.896 |
|  | 2-point swallow tail sign scale | 2 | 80.8 | 83.3 | 4.85 | 0.23 | 80.8 | 83.3 | 82.1 | 0.872 |
| PSP vs. HC | bilaterally detected ^b,*^ | - | 88.2 | 100 | >10 | 0.12 | 100 | 93.8 | 95.7 | - |
|  | unilaterally detected ^b,*^ | - | 85.3 | 100 | >10 | 0.15 | 100 | 92.3 | 94.7 | - |
|  | bilaterally normal | - | 97.1 | 68.3 | 3.07 | 0.04 | 63.5 | 97.6 | 78.7 | - |
|  | unilaterally normal ^b^ | - | 97.1 | 83.3 | 5.82 | 0.04 | 76.7 | 98.0 | 88.3 | - |
|  | 6-point visibility scale ^a,*^ | 5 | 97.1 | 100 | >10 | 0.03 | 100 | 98.4 | 98.9 | 0.981 |
|  | 2-point area of hyperintensity scale ^a,*^ | 0 | 97.1 | 100 | >10 | 0.03 | 100 | 98.4 | 98.9 | 0.981 |
|  | 2-point swallow tail sign scale ^b,*^ | 1 | 88.2 | 100 | >10 | 0.12 | 100 | 93.8 | 95.7 | 0.973 |
| iRBD vs. HC | bilaterally detected | - | - | - | - | - | - | - | - | - |
|  | unilaterally detected | - | - | - | - | - | - | - | - | - |
|  | bilaterally normal | - | 83.9 | 68.3 | 2.65 | 0.24 | 57.8 | 89.1 | 73.6 | - |
|  | unilaterally normal | - | 74.2 | 83.3 | 4.45 | 0.31 | 69.7 | 86.2 | 80.2 | - |
|  | 6-point visibility scale | - | - | - | - | - | - | - | - | - |
|  | 2-point area of hyperintensity scale | 2 | 74.2 | 81.7 | 4.05 | 0.32 | 67.6 | 86.0 | 79.1 | 0.846 |
|  | 2-point swallow tail sign scale | - | - | - | - | - | - | - | - | - |
| Early-stage PD vs. MSA-C | bilaterally detected | - | 77.4 | 78.8 | 3.66 | 0.29 | 81.4 | 74.5 | 78.1 | - |
|  | unilaterally detected | - | 62.9 | 86.5 | 4.67 | 0.43 | 84.8 | 66.2 | 73.7 | - |
|  | bilaterally normal | - | - | - | - | - | - | - | - | - |
|  | unilaterally normal | - | 100 | 19.2 | 1.24 | 0 | 59.6 | 100 | 63.2 | - |
|  | 6-point visibility scale ^b^ | 4 | 88.7 | 84.5 | 5.77 | 0.13 | 87.3 | 86.3 | 86.8 | 0.882 |
|  | 2-point area of hyperintensity scale | 0 | 96.8 | 55.8 | 2.19 | 0.06 | 72.3 | 93.5 | 78.1 | 0.770 |
|  | 2-point swallow tail sign scale | 1 | 77.4 | 78.8 | 3.66 | 0.29 | 81.4 | 74.5 | 78.1 | 0.817 |
| Early-stage PD vs. iRBD | bilaterally detected | - | 77.4 | 93.5 | 12 | 0.24 | 96.0 | 67.4 | 82.8 | - |
|  | unilaterally detected | - | 62.9 | 93.5 | 9.75 | 0.4 | 95.1 | 55.8 | 73.1 | - |
|  | bilaterally normal | - | 100 | 16.1 | 1.19 | 0 | 70.5 | 100 | 72.0 | - |
|  | unilaterally normal | - | 100 | 25.8 | 1.35 | 0 | 72.9 | 100 | 75.3 | - |
|  | 6-point visibility scale ^b^ | 4 | 88.7 | 90.3 | 9.17 | 0.13 | 94.8 | 80.0 | 89.2 | 0.937 |
|  | 2-point area of hyperintensity scale | 0 | 96.8 | 64.5 | 2.73 | 0.05 | 84.5 | 90.9 | 86.0 | 0.814 |
|  | 2-point swallow tail sign scale | 1 | 77.4 | 93.5 | 12 | 0.24 | 96.0 | 67.4 | 82.8 | 0.879 |
| MSA-P vs. MSA-C | bilaterally detected | - | 94.3 | 78.8 | 4.46 | 0.07 | 82.0 | 93.2 | 86.7 | - |
|  | unilaterally detected ^b^ | - | 83.0 | 86.5 | 6.17 | 0.2 | 86.3 | 83.3 | 84.8 | - |
|  | bilaterally normal | - | - | - | - | - | - | - | - | - |
|  | unilaterally normal | - | 100 | 19.2 | 1.24 | 0 | 55.8 | 100 | 60.0 | - |
|  | 6-point visibility scale ^b^ | 4 | 94.3 | 84.5 | 6.13 | 0.07 | 86.2 | 93.6 | 89.5 | 0.909 |
|  | 2-point area of hyperintensity scale | 0 | 98.1 | 55.8 | 2.22 | 0.03 | 69.3 | 96.7 | 77.1 | 0.773 |
|  | 2-point swallow tail sign scale | 1 | 94.3 | 78.8 | 4.46 | 0.07 | 82.0 | 93.2 | 86.7 | 0.896 |
| PSP vs. MSA-C | bilaterally detected | - | 88.2 | 78.8 | 4.17 | 0.15 | 73.2 | 91.1 | 82.6 | - |
|  | unilaterally detected ^b^ | - | 85.3 | 86.5 | 6.34 | 0.17 | 80.6 | 90.0 | 86.0 | - |
|  | bilaterally normal | - | - | - | - | - | - | - | - | - |
|  | unilaterally normal | - | - | - | - | - | - | - | - | - |
|  | 6-point visibility scale ^b^ | 4 | 94.1 | 84.5 | 6.12 | 0.07 | 80.0 | 95.7 | 88.4 | 0.895 |
|  | 2-point area of hyperintensity scale | 0 | 97.1 | 55.8 | 2.19 | 0.05 | 58.9 | 96.7 | 72.1 | 0.757 |
|  | 2-point swallow tail sign scale ^b^ | 0 | 85.3 | 86.5 | 6.34 | 0.17 | 80.6 | 90.0 | 86.0 | 0.864 |
| MSA-P vs. iRBD | bilaterally detected ^a^ | - | 94.3 | 93.5 | 14.62 | 0.06 | 96.2 | 90.6 | 94.0 | - |
|  | unilaterally detected ^b^ | - | 83.0 | 93.5 | 12.87 | 0.18 | 95.7 | 76.3 | 86.9 | - |
|  | bilaterally normal | - | - | - | - | - | - | - | - | - |
|  | unilaterally normal | - | 100 | 25.8 | 1.35 | 0 | 69.7 | 100 | 72.6 | - |
|  | 6-point visibility scale ^b^ | 4 | 94.3 | 90.3 | 9.75 | 0.06 | 94.3 | 90.3 | 92.9 | 0.953 |
|  | 2-point area of hyperintensity scale | 0 | 98.1 | 64.5 | 2.77 | 0.03 | 82.5 | 95.2 | 85.7 | 0.818 |
|  | 2-point swallow tail sign scale ^a^ | 1 | 94.3 | 93.5 | 14.62 | 0.06 | 96.2 | 90.6 | 94.0 | 0.943 |
| PSP vs. iRBD | bilaterally detected ^b^ | - | 88.2 | 93.5 | 13.68 | 0.13 | 93.8 | 87.9 | 90.8 | - |
|  | unilaterally detected ^b^ | - | 85.3 | 93.5 | 13.22 | 0.16 | 93.5 | 85.3 | 89.2 | - |
|  | bilaterally normal | - | - | - | - | - | - | - | - | - |
|  | unilaterally normal | - | - | - | - | - | - | - | - | - |
|  | 6-point visibility scale ^b^ | 4 | 94.1 | 90.3 | 9.73 | 0.07 | 91.4 | 93.3 | 92.3 | 0.935 |
|  | 2-point area of hyperintensity scale | 0 | 97.1 | 64.5 | 2.74 | 0.05 | 75.0 | 95.2 | 81.5 | 0.801 |
|  | 2-point swallow tail sign scale ^b^ | 1 | 88.2 | 93.5 | 13.68 | 0.13 | 93.8 | 87.9 | 90.8 | 0.908 |
| Advanced PD vs. MSA-C | bilaterally detected ^b^ | - | 96.3 | 78.8 | 4.55 | 0.05 | 82.5 | 95.3 | 87.7 | - |
|  | unilaterally detected ^b^ | - | 87.0 | 86.5 | 6.47 | 0.15 | 87.0 | 86.5 | 86.8 | - |
|  | bilaterally normal | - | - | - | - | - | - | - | - | - |
|  | unilaterally normal | - | 100 | 19.2 | 1.24 | 0 | 56.3 | 100 | 60.4 | - |
|  | 6-point visibility scale ^b^ | 4 | 98.2 | 84.5 | 6.38 | 0.02 | 86.9 | 97.8 | 91.5 | 0.922 |
|  | 2-point area of hyperintensity scale | 0 | 98.2 | 55.8 | 2.22 | 0.03 | 69.7 | 96.7 | 77.4 | 0.774 |
|  | 2-point swallow tail sign scale | 1 | 96.3 | 78.8 | 4.55 | 0.05 | 82.5 | 95.3 | 87.7 | 0.907 |
| Advanced PD vs. iRBD | bilaterally detected ^a^ | - | 96.3 | 93.5 | 14.93 | 0.04 | 96.3 | 93.5 | 95.3 | - |
|  | unilaterally detected ^b^ | - | 87.0 | 93.5 | 13.49 | 0.14 | 95.9 | 80.6 | 89.4 | - |
|  | bilaterally normal | - | 100 | 16.1 | 1.19 | 0 | 67.5 | 100 | 69.4 | - |
|  | unilaterally normal | - | 100 | 25.8 | 1.35 | 0 | 70.1 | 100 | 72.9 | - |
|  | 6-point visibility scale ^a^ | 4 | 98.2 | 90.3 | 10.14 | 0.02 | 94.6 | 96.6 | 95.3 | 0.961 |
|  | 2-point area of hyperintensity scale | 0 | 98.2 | 64.5 | 2.77 | 0.03 | 82.8 | 95.2 | 85.9 | 0.818 |
|  | 2-point swallow tail sign scale ^a^ | 1 | 96.3 | 93.5 | 14.93 | 0.04 | 96.3 | 93.5 | 95.3 | 0.951 |

^a^ DNH assessment method with LR+ > 10 and LR- < 0.1.

^b^ DNH assessment method with 5 ≤ LR+ ≤ 10 and 0.1 < LR- < 0.2, or LR+ > 10 and 0.1 < LR- < 0.2, or 5 ≤ LR+ ≤ 10 and LR- < 0.1.

^*^ DNH assessment method with higher diagnostic accuracy in those with LR+ ≥ 5 and LR- ≤ 0.2.

DNH, dorsal nigral hyperintensity; PD, Parkinson’s disease; iRBD, idiopathic rapid eye movement sleep behavior disorder; MSA, multiple system atrophy; MSA-P, MSA-parkinsonian type; MSA-C, MSA-cerebellar type; PSP, progressive supranuclear palsy; HC, healthy control; LR+, positive likelihood ratio; LR-, negative likelihood ratio; PPV, positive predictive value; NPV, negative predictive value; AUC, area under the receiver operating characteristic curve.

**Table S8 Diagnostic and differential diagnostic performances of the seven DNH assessment methods in the pooled dataset**

|  | Methods | DNH score | Sensitivity (%) | Specificity (%) | LR+ | LR- | PPV (%) | NPV (%) | Accuracy (%) | AUC |
| --- | --- | --- | --- | --- | --- | --- | --- | --- | --- | --- |
| PD vs. HC | bilaterally detected ^b^ | - | 85.2 | 100 | >10 | 0.15 | 100 | 80.6 | 90.8 | - |
|  | unilaterally detected | - | 72.8 | 100 | >10 | 0.27 | 100 | 69.4 | 83.2 | - |
|  | bilaterally normal | - | 100 | 66.0 | 2.94 | 0 | 82.7 | 100 | 87.0 | - |
|  | unilaterally normal ^b^ | - | 100 | 83.0 | 5.88 | 0 | 90.5 | 100 | 93.5 | - |
|  | 6-point visibility scale ^a,*^ | 6 | 100 | 100 | >10 | 0 | 100 | 100 | 100 | 1.000 |
|  | 2-point area of hyperintensity scale ^a,*^ | 1 | 100 | 98.0 | 50 | 0 | 98.8 | 100 | 99.2 | 1.000 |
|  | 2-point swallow tail sign scale ^b^ | 1 | 85.2 | 100 | >10 | 0.15 | 100 | 80.6 | 90.8 | 0.987 |
| Early-stage PD vs. HC | bilaterally detected | - | 79.6 | 100 | >10 | 0.2 | 100 | 82.0 | 89.4 | - |
|  | unilaterally detected | - | 65.7 | 100 | >10 | 0.34 | 100 | 73.0 | 82.2 | - |
|  | bilaterally normal | - | 100 | 66.0 | 2.94 | 0 | 76.1 | 100 | 83.7 | - |
|  | unilaterally normal ^b^ | - | 100 | 83.0 | 5.88 | 0 | 86.4 | 100 | 91.8 | - |
|  | 6-point visibility scale ^a,*^ | 6 | 100 | 100 | >10 | 0 | 100 | 100 | 100 | 1.000 |
|  | 2-point area of hyperintensity scale ^a,*^ | 1 | 100 | 98.0 | 50 | 0 | 98.2 | 100 | 99.0 | 1.000 |
|  | 2-point swallow tail sign scale ^b^ | 2 | 100 | 83.0 | 5.88 | 0 | 86.4 | 100 | 91.8 | 0.983 |
| Advanced PD vs. HC | bilaterally detected ^a,*^ | - | 96.3 | 100 | >10 | 0.04 | 100 | 98.0 | 98.7 | - |
|  | unilaterally detected ^b,*^ | - | 87.0 | 100 | >10 | 0.13 | 100 | 93.5 | 95.5 | - |
|  | bilaterally normal | - | 100 | 66.0 | 2.94 | 0 | 61.4 | 100 | 77.9 | - |
|  | unilaterally normal ^b^ | - | 100 | 83.0 | 5.88 | 0 | 76.1 | 100 | 89.0 | - |
|  | 6-point visibility scale ^a,*^ | 5 | 100 | 100 | >10 | 0 | 100 | 100 | 100 | 1.000 |
|  | 2-point area of hyperintensity scale ^a,*^ | 0 | 98.2 | 100 | >10 | 0.02 | 100 | 99.0 | 99.4 | 1.000 |
|  | 2-point swallow tail sign scale ^a,*^ | 1 | 96.3 | 100 | >10 | 0.04 | 100 | 98.0 | 98.7 | 0.997 |
| MSA-P vs. HC | bilaterally detected ^a,*^ | - | 94.3 | 100 | >10 | 0.06 | 100 | 95.2 | 97.3 | - |
|  | unilaterally detected ^b^ | - | 83.9 | 100 | >10 | 0.16 | 100 | 87.7 | 92.5 | - |
|  | bilaterally normal | - | 100 | 66.0 | 2.94 | 0 | 71.9 | 100 | 81.8 | - |
|  | unilaterally normal ^b^ | - | 100 | 83.0 | 5.88 | 0 | 83.7 | 100 | 90.9 | - |
|  | 6-point visibility scale ^a,*^ | 6 | 100 | 100 | >10 | 0 | 100 | 100 | 100 | 1.000 |
|  | 2-point area of hyperintensity scale ^a,*^ | 1 | 100 | 98.0 | 50 | 0 | 97.8 | 100 | 98.9 | 1.000 |
|  | 2-point swallow tail sign scale ^a^ | 1 | 94.3 | 100 | >10 | 0.06 | 100 | 95.2 | 97.3 | 0.995 |
| MSA-C vs. HC | bilaterally detected | - | 20.5 | 100 | >10 | 0.8 | 100 | 58.8 | 62.8 | - |
|  | unilaterally detected | - | 13.6 | 100 | >10 | 0.86 | 100 | 56.8 | 59.6 | - |
|  | bilaterally normal | - | 92 | 66.0 | 2.71 | 0.12 | 70.4 | 90.4 | 78.2 | - |
|  | unilaterally normal | - | 79.5 | 83.0 | 4.68 | 0.25 | 80.5 | 82.2 | 81.4 | - |
|  | 6-point visibility scale | 8 | 79.5 | 82.0 | 4.42 | 0.25 | 79.5 | 82.0 | 80.9 | 0.892 |
|  | 2-point area of hyperintensity scale | 2 | 79.5 | 82.0 | 4.42 | 0.25 | 79.5 | 82.0 | 80.9 | 0.892 |
|  | 2-point swallow tail sign scale | 2 | 79.5 | 83.0 | 4.68 | 0.25 | 80.5 | 82.2 | 81.4 | 0.864 |
| PSP vs. HC | bilaterally detected ^b,*^ | - | 88.2 | 100 | >10 | 0.12 | 100 | 96.2 | 97.0 | - |
|  | unilaterally detected ^b,*^ | - | 85.3 | 100 | >10 | 0.15 | 100 | 95.2 | 96.3 | - |
|  | bilaterally normal | - | 97.1 | 66.0 | 2.85 | 0.04 | 49.3 | 98.5 | 73.9 | - |
|  | unilaterally normal ^b^ | - | 97.1 | 83.0 | 5.71 | 0.04 | 66.0 | 98.8 | 86.6 | - |
|  | 6-point visibility scale ^a,*^ | 5 | 97.1 | 100 | >10 | 0.03 | 100 | 99.0 | 99.3 | 0.980 |
|  | 2-point area of hyperintensity scale ^a,*^ | 0 | 97.1 | 100 | >10 | 0.03 | 100 | 99.0 | 99.3 | 0.980 |
|  | 2-point swallow tail sign scale ^b,*^ | 1 | 88.2 | 100 | >10 | 0.12 | 100 | 96.2 | 97.0 | 0.973 |
| iRBD vs. HC | bilaterally detected | - | 6.5 | 100 | >10 | 0.94 | 100 | 77.5 | 77.9 | - |
|  | unilaterally detected | - | 6.5 | 100 | >10 | 0.94 | 100 | 77.5 | 77.9 | - |
|  | bilaterally normal | - | 83.9 | 66.0 | 2.47 | 0.24 | 43.3 | 93.0 | 70.2 | - |
|  | unilaterally normal | - | 74.2 | 83.0 | 4.36 | 0.31 | 57.5 | 91.2 | 80.9 | - |
|  | 6-point visibility scale | 8 | 74.2 | 82.0 | 4.12 | 0.31 | 56.1 | 91.1 | 80.2 | 0.843 |
|  | 2-point area of hyperintensity scale | 2 | 74.2 | 82.0 | 4.12 | 0.31 | 56.1 | 91.1 | 80.2 | 0.844 |
|  | 2-point swallow tail sign scale | 2 | 74.2 | 83.0 | 4.36 | 0.31 | 57.5 | 91.2 | 80.9 | 0.810 |
| Early-stage PD vs. MSA-C | bilaterally detected | - | 79.6 | 79.5 | 3.89 | 0.26 | 82.7 | 76.1 | 79.6 | - |
|  | unilaterally detected | - | 65.7 | 86.4 | 4.82 | 0.4 | 85.5 | 67.3 | 75.0 | - |
|  | bilaterally normal | - | 100 | 8.0 | 1.09 | 0 | 57.1 | 100 | 58.7 | - |
|  | unilaterally normal | - | 100 | 20.5 | 1.26 | 0 | 60.7 | 100 | 64.3 | - |
|  | 6-point visibility scale ^b^ | 4 | 88.0 | 84.1 | 5.53 | 0.14 | 87.2 | 85.1 | 86.2 | 0.892 |
|  | 2-point area of hyperintensity scale | 0 | 96.3 | 55.7 | 2.17 | 0.07 | 72.7 | 92.5 | 78.1 | 0.768 |
|  | 2-point swallow tail sign scale | 1 | 79.6 | 80.7 | 4.12 | 0.25 | 83.5 | 76.3 | 80.1 | 0.836 |
| Early-stage PD vs. iRBD | bilaterally detected | - | 79.6 | 93.5 | 12.34 | 0.22 | 97.7 | 56.9 | 82.7 | - |
|  | unilaterally detected | - | 65.7 | 93.5 | 10.19 | 0.37 | 97.3 | 43.9 | 71.9 | - |
|  | bilaterally normal | - | 100 | 16.1 | 1.19 | 0 | 80.6 | 100 | 81.3 | - |
|  | unilaterally normal | - | 100 | 25.8 | 1.35 | 0 | 82.4 | 100 | 83.5 | - |
|  | 6-point visibility scale ^b^ | 5 | 93.5 | 87.1 | 7.25 | 0.07 | 96.2 | 79.4 | 92.1 | 0.940 |
|  | 2-point area of hyperintensity scale | 0 | 96.3 | 64.5 | 2.71 | 0.06 | 90.4 | 83.3 | 89.2 | 0.813 |
|  | 2-point swallow tail sign scale | 1 | 79.6 | 93.5 | 12.34 | 0.22 | 97.7 | 56.9 | 82.7 | 0.888 |
| MSA-P vs. MSA-C | bilaterally detected | - | 94.3 | 79.5 | 4.61 | 0.07 | 82.0 | 93.3 | 86.9 | - |
|  | unilaterally detected ^b^ | - | 83.9 | 86.4 | 6.15 | 0.19 | 85.9 | 84.4 | 85.1 | - |
|  | bilaterally normal | - | 100 | 8.0 | 1.09 | 0 | 51.8 | 100 | 53.7 | - |
|  | unilaterally normal | - | 100 | 20.5 | 1.26 | 0 | 55.4 | 100 | 60.0 | - |
|  | 6-point visibility scale ^b^ | 4 | 94.3 | 84.1 | 5.92 | 0.07 | 85.4 | 93.7 | 89.1 | 0.913 |
|  | 2-point area of hyperintensity scale | 0 | 97.7 | 55.7 | 2.2 | 0.04 | 68.5 | 96.1 | 76.6 | 0.772 |
|  | 2-point swallow tail sign scale | 1 | 94.3 | 80.7 | 4.88 | 0.07 | 82.8 | 93.4 | 87.4 | 0.903 |
| PSP vs. MSA-C | bilaterally detected | - | 88.2 | 79.5 | 4.31 | 0.15 | 62.5 | 94.6 | 82.0 | - |
|  | unilaterally detected ^b^ | - | 85.3 | 86.4 | 6.25 | 0.17 | 70.7 | 93.8 | 86.1 | - |
|  | bilaterally normal | - | 97.1 | 8.0 | 1.05 | 0.37 | 28.9 | 87.5 | 32.8 | - |
|  | unilaterally normal | - | 97.1 | 20.5 | 1.22 | 0.14 | 32.0 | 94.7 | 41.8 | - |
|  | 6-point visibility scale ^b^ | 4 | 94.1 | 84.1 | 5.92 | 0.07 | 69.6 | 97.4 | 86.9 | 0.900 |
|  | 2-point area of hyperintensity scale | 0 | 97.1 | 55.7 | 2.19 | 0.05 | 45.8 | 98.0 | 67.2 | 0.757 |
|  | 2-point swallow tail sign scale ^b^ | 0 | 85.3 | 87.5 | 6.82 | 0.17 | 72.5 | 93.9 | 86.9 | 0.870 |
| MSA-P vs. iRBD | bilaterally detected ^a,*^ | - | 94.3 | 93.5 | 14.61 | 0.06 | 97.6 | 85.3 | 94.1 | - |
|  | unilaterally detected ^b^ | - | 83.9 | 93.5 | 13.01 | 0.17 | 97.3 | 67.4 | 86.4 | - |
|  | bilaterally normal | - | 100 | 16.1 | 1.19 | 0 | 77.0 | 100 | 78.0 | - |
|  | unilaterally normal | - | 100 | 25.8 | 1.35 | 0 | 79.1 | 100 | 80.5 | - |
|  | 6-point visibility scale ^b,*^ | 4 | 94.3 | 90.3 | 9.74 | 0.06 | 96.5 | 84.8 | 93.2 | 0.953 |
|  | 2-point area of hyperintensity scale | 0 | 97.7 | 64.5 | 2.75 | 0.04 | 88.5 | 90.9 | 89.0 | 0.817 |
|  | 2-point swallow tail sign scale ^a,*^ | 1 | 94.3 | 93.5 | 14.61 | 0.06 | 97.6 | 85.3 | 94.1 | 0.943 |
| Advanced PD vs. MSA-C | bilaterally detected | - | 96.3 | 79.5 | 4.71 | 0.05 | 74.3 | 97.2 | 85.9 | - |
|  | unilaterally detected ^b^ | - | 87.0 | 86.4 | 6.38 | 0.15 | 79.7 | 91.6 | 86.6 | - |
|  | bilaterally normal | - | 100 | 8.0 | 1.09 | 0 | 40.0 | 100 | 43.0 | - |
|  | unilaterally normal | - | 100 | 20.5 | 1.26 | 0 | 43.5 | 100 | 50.7 | - |
|  | 6-point visibility scale ^b^ | 4 | 98.2 | 84.1 | 6.17 | 0.02 | 79.1 | 98.7 | 89.4 | 0.926 |
|  | 2-point area of hyperintensity scale | 0 | 98.2 | 55.7 | 2.21 | 0.03 | 57.6 | 98.0 | 71.8 | 0.773 |
|  | 2-point swallow tail sign scale | 1 | 96.3 | 80.7 | 4.98 | 0.05 | 75.4 | 97.3 | 86.6 | 0.913 |

^a^ DNH assessment method with LR+ > 10 and LR- < 0.1.

^b^ DNH assessment method with 5 ≤ LR+ ≤ 10 and 0.1 < LR- < 0.2, or LR+ > 10 and 0.1 < LR- < 0.2, or 5 ≤ LR+ ≤ 10 and LR- < 0.1.

^*^ DNH assessment method with higher diagnostic accuracy in those with LR+ ≥ 5 and LR- ≤ 0.2.

DNH, dorsal nigral hyperintensity; PD, Parkinson’s disease; iRBD, idiopathic rapid eye movement sleep behavior disorder; MSA, multiple system atrophy; MSA-P, MSA-parkinsonian type; MSA-C, MSA-cerebellar type; PSP, progressive supranuclear palsy; HC, healthy control; LR+, positive likelihood ratio; LR-, negative likelihood ratio; PPV, positive predictive value; NPV, negative predictive value; AUC, area under the receiver operating characteristic curve.

**Table S9 Validation of diagnostic and differential diagnostic performances of the DNH abnormality**

|  | Methods ^a^ | DNH score ^a^ | Sensitivity (%) | Specificity (%) | PPV (%) | NPV (%) | Accuracy (%) |
| --- | --- | --- | --- | --- | --- | --- | --- |
| Early-stage PD vs. HC | 6-point visibility scale | 6 | 100 | 100 | 100 | 100 | 100 |
|  | 2-point area of hyperintensity scale | 1 | 100 | 97.5 | 97.9 | 100 | 98.8 |
| MSA-P vs. HC | bilaterally detected | - | 94.1 | 100 | 100 | 95.2 | 97.3 |
|  | 6-point visibility scale | 6 | 100 | 100 | 100 | 100 | 100 |
|  | 2-point area of hyperintensity scale | 1 | 100 | 97.5 | 97.1 | 100 | 98.6 |
|  | 2-point swallow tail sign scale | 1 | 94.1 | 100 | 100 | 95.2 | 97.3 |
| Early-stage PD vs. MSA-C | 6-point visibility scale | 4 | 87.0 | 83.3 | 87.0 | 83.3 | 85.4 |
| MSA-P vs. MSA-C | unilaterally detected | - | 85.3 | 86.1 | 85.3 | 86.1 | 85.7 |
|  | 6-point visibility scale | 4 | 94.1 | 83.3 | 84.2 | 93.8 | 88.6 |

^a^ The optimal DNH assessment method and DNH score in the development cohort.

PD, Parkinson’s disease; MSA, multiple system atrophy; MSA-P, MSA-parkinsonian type; MSA-C, MSA-cerebellar type; HC, healthy control; DNH, dorsal nigral hyperintensity; PPV, positive predictive value; NPV, negative predictive value.

**Table S10 Subgroup analyses of diagnostic and differential diagnostic performances of the seven DNH assessment methods in age- and education-matched participants aged over 60**

|  | Methods | DNH score | Sensitivity (%) | Specificity (%) | LR+ | LR- | PPV (%) | NPV (%) | Accuracy (%) | AUC |
| --- | --- | --- | --- | --- | --- | --- | --- | --- | --- | --- |
| Advanced PD vs. HC | bilaterally detected ^a,*^ | - | 94.6 | 100 | >10 | 0.05 | 100 | 95.7 | 97.6 | - |
|  | unilaterally detected ^b,*^ | - | 83.8 | 100 | >10 | 0.16 | 100 | 88.2 | 92.7 | - |
|  | bilaterally normal | - | 100 | 62.2 | 2.65 | 0 | 68.5 | 100 | 79.3 | - |
|  | unilaterally normal | - | 100 | 77.8 | 4.50 | 0 | 78.7 | 100 | 87.8 | - |
|  | 6-point visibility scale ^a,*^ | 5 | 100 | 100 | >10 | 0 | 100 | 100 | 100 | 1.000 |
|  | 2-point area of hyperintensity scale ^a,*^ | 1 | 100 | 97.8 | 45 | 0 | 97.4 | 100 | 98.8 | 1.000 |
|  | 2-point swallow tail sign scale ^a,*^ | 1 | 94.6 | 100 | >10 | 0.05 | 100 | 95.7 | 97.6 | 0.994 |
| MSA-P vs. HC | bilaterally detected ^a,*^ | - | 92.5 | 100 | >10 | 0.08 | 100 | 93.8 | 96.5 | - |
|  | unilaterally detected ^a^ | - | 82.5 | 100 | >10 | 0.18 | 100 | 86.5 | 91.8 | - |
|  | bilaterally normal | - | 100 | 62.2 | 2.65 | 0 | 70.2 | 100 | 80.0 | - |
|  | unilaterally normal ^b^ | - | 100 | 77.8 | 4.5 | 0 | 80 | 100 | 88.2 | - |
|  | 6-point visibility scale ^a,*^ | 6 | 100 | 100 | >10 | 0 | 100 | 100 | 100 | 1.000 |
|  | 2-point area of hyperintensity scale ^a,*^ | 1 | 100 | 97.8 | 45 | 0 | 97.6 | 100 | 98.8 | 1.000 |
|  | 2-point swallow tail sign scale ^a,*^ | 1 | 92.5 | 100 | >10 | 0.08 | 100 | 93.8 | 96.5 | 0.992 |
| MSA-C vs. HC | bilaterally detected | - | 18.2 | 100 | >10 | 0.82 | 100 | 62.5 | 65.4 | - |
|  | unilaterally detected | - | 12.1 | 100 | >10 | 0.88 | 100 | 60.8 | 62.8 | - |
|  | bilaterally normal | - | 93.9 | 62.2 | 2.49 | 0.10 | 64.6 | 93.3 | 75.6 | - |
|  | unilaterally normal | - | 75.8 | 77.8 | 3.41 | 0.31 | 71.4 | 81.4 | 76.9 | - |
|  | 6-point visibility scale | 8 | 75.8 | 77.8 | 3.41 | 0.31 | 71.4 | 81.4 | 76.9 | 0.880 |
|  | 2-point area of hyperintensity scale | 2 | 75.8 | 77.8 | 3.41 | 0.31 | 71.4 | 81.4 | 76.9 | 0.883 |
|  | 2-point swallow tail sign scale | 2 | 75.8 | 77.8 | 3.41 | 0.31 | 71.4 | 81.4 | 76.9 | 0.842 |
| PSP vs. HC | bilaterally detected ^b,*^ | - | 85.7 | 100 | >10 | 0.14 | 100 | 91.8 | 94.5 | - |
|  | unilaterally detected ^b,*^ | - | 82.1 | 100 | >10 | 0.18 | 100 | 90 | 93.2 | - |
|  | bilaterally normal | - | 96.4 | 62.2 | 2.55 | 0.06 | 61.4 | 96.6 | 75.3 | - |
|  | unilaterally normal | - | 96.4 | 77.8 | 4.34 | 0.05 | 73 | 97.2 | 84.9 | - |
|  | 6-point visibility scale ^a,*^ | 5 | 96.4 | 100 | >10 | 0.04 | 100 | 97.8 | 98.6 | 0.975 |
|  | 2-point area of hyperintensity scale ^a,*^ | 0 | 96.4 | 100 | >10 | 0.04 | 100 | 97.8 | 98.6 | 0.976 |
|  | 2-point swallow tail sign scale ^b,*^ | 1 | 85.7 | 100 | >10 | 0.14 | 100 | 91.8 | 94.5 | 0.964 |
| Advanced PD vs. MSA-C | bilaterally detected ^b^ | - | 94.6 | 81.8 | 5.20 | 0.07 | 85.4 | 93.1 | 88.6 | - |
|  | unilaterally detected ^b^ | - | 83.8 | 87.9 | 6.91 | 0.18 | 88.6 | 82.9 | 85.7 | - |
|  | bilaterally normal | - | - | - | - | - | - | - | - | - |
|  | unilaterally normal | - | 100 | 24.2 | 1.32 | 0 | 59.7 | 100 | 64.3 | - |
|  | 6-point visibility scale ^b^ | 4 | 97.3 | 81.8 | 5.35 | 0.03 | 85.7 | 96.4 | 90.0 | 0.924 |
|  | 2-point area of hyperintensity scale | 0 | 97.3 | 54.5 | 2.14 | 0.05 | 70.6 | 94.7 | 77.1 | 0.765 |
|  | 2-point swallow tail sign scale ^b^ | 1 | 94.6 | 81.8 | 5.20 | 0.07 | 85.4 | 93.1 | 88.6 | 0.907 |
| MSA-P vs. MSA-C | bilaterally detected ^b^ | - | 92.5 | 81.8 | 5.09 | 0.09 | 86 | 90 | 87.7 | - |
|  | unilaterally detected ^b^ | - | 82.5 | 87.9 | 6.81 | 0.20 | 89.2 | 80.6 | 84.9 | - |
|  | bilaterally normal | - | - | - | - | - | - | - | - | - |
|  | unilaterally normal | - | 100 | 24.2 | 1.32 | 0 | 61.5 | 100 | 65.8 | - |
|  | 6-point visibility scale ^b^ | 4 | 92.5 | 81.8 | 5.09 | 0.09 | 86 | 90 | 87.7 | 0.906 |
|  | 2-point area of hyperintensity scale | 0 | 97.5 | 54.5 | 2.15 | 0.05 | 72.2 | 94.7 | 78.1 | 0.766 |
|  | 2-point swallow tail sign scale ^b^ | 1 | 92.5 | 81.8 | 5.09 | 0.09 | 86 | 90 | 87.7 | 0.897 |
| PSP vs. MSA-C | bilaterally detected | - | 85.7 | 81.8 | 4.71 | 0.17 | 80 | 87.1 | 83.6 | - |
|  | unilaterally detected ^b^ | - | 82.1 | 87.9 | 6.78 | 0.20 | 85.2 | 85.3 | 85.2 | - |
|  | bilaterally normal | - | - | - | - | - | - | - | - | - |
|  | unilaterally normal | - | - | - | - | - | - | - | - | - |
|  | 6-point visibility scale ^b^ | 4 | 92.9 | 81.8 | 5.11 | 0.09 | 81.3 | 93.1 | 86.9 | 0.891 |
|  | 2-point area of hyperintensity scale | 0 | 96.4 | 54.5 | 2.12 | 0.07 | 64.3 | 94.7 | 73.8 | 0.746 |
|  | 2-point swallow tail sign scale ^b^ | 0 | 82.1 | 87.9 | 6.78 | 0.20 | 85.2 | 85.3 | 85.2 | 0.860 |

^a^ DNH assessment method with LR+ > 10 and LR- < 0.1.

^b^ DNH assessment method with 5 ≤ LR+ ≤ 10 and 0.1 < LR- < 0.2, or LR+ > 10 and 0.1 < LR- < 0.2, or 5 ≤ LR+ ≤ 10 and LR- < 0.1.

^*^ DNH assessment method with higher diagnostic accuracy in those with LR+ ≥ 5 and LR- ≤ 0.2.

DNH, dorsal nigral hyperintensity; PD, Parkinson’s disease; MSA, multiple system atrophy; MSA-P, MSA-parkinsonian type; MSA-C, MSA-cerebellar type; PSP, progressive supranuclear palsy; HC, healthy control; LR+, positive likelihood ratio; LR-, negative likelihood ratio; PPV, positive predictive value; NPV, negative predictive value; AUC, area under the receiver operating characteristic curve.

The advanced PD (*n =* 37), MSA-P (*n =* 40), MSA-C (*n =* 33), PSP (*n =* 28), and HC (*n =* 45) subgroups aged over 60 were matched for age (*p* = 0.115) and years of education (*p* = 0.085).

|  | 2-year follow-up | 1-year follow-up | ＜1-year follow-up |
| --- | --- | --- | --- |
|  | *n =* 7 | *n =* 16 | *n =* 8 |
| Baseline characteristics |  |  |  |
| Age, year, median (IQR) | 64.0 (51.0, 65.0) | 63.5 (58.3, 71.5) | 65.5 (58.8, 68.0) |
| Sex, male/female | 6/1 | 9/7 | 5/3 |
| Disease duration, year, median (IQR) | 3.0 (1.0, 5.0) | 3.0 (1.3, 5.8) | 3.0 (1.3, 4.5) |
| Parkinson’s disease at follow-up, n | 1 | 1 | 0 |

**Table S11 Follow-up evaluations of iRBD patients**

iRBD, idiopathic rapid eye movement sleep behavior disorder; DNH, dorsal nigral hyperintensity; n, number; IQR, interquartile range.

**Fig. S1** Flowchart of participant inclusion


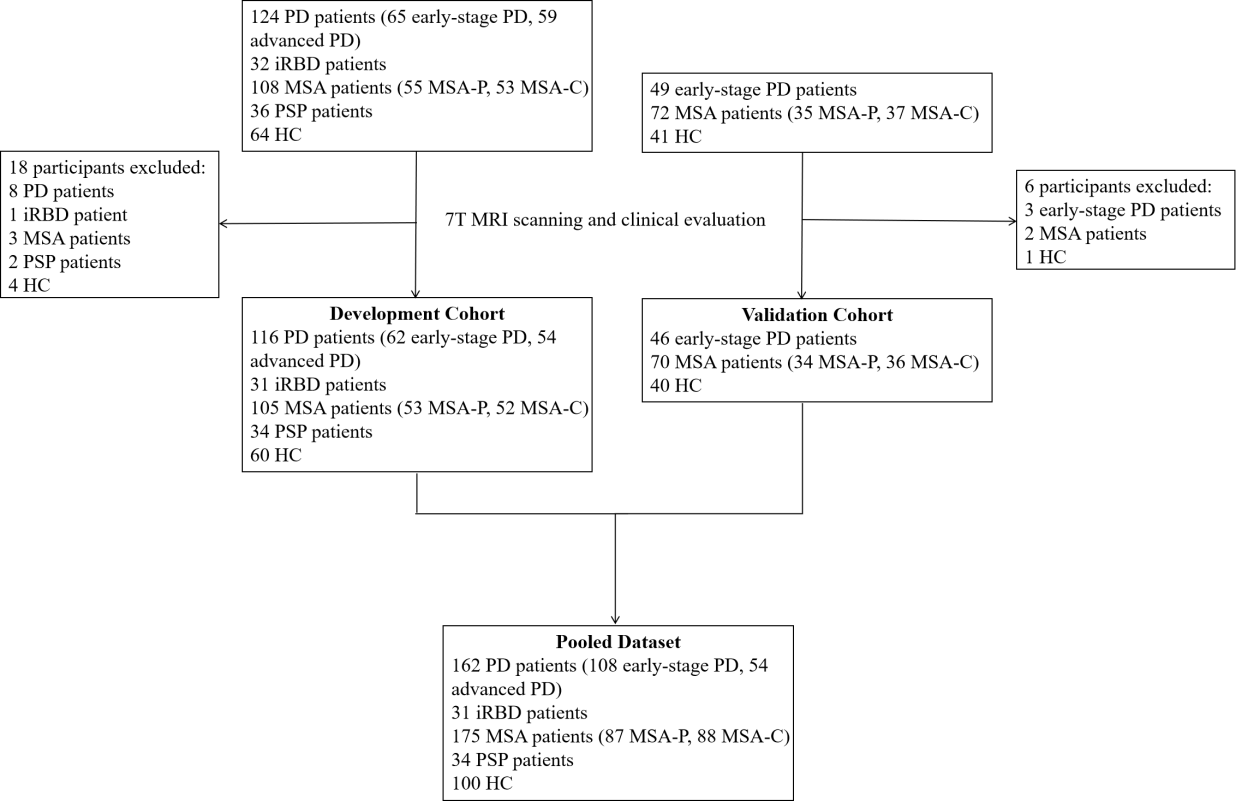


PD, Parkinson’s disease; iRBD, idiopathic rapid eye movement sleep behavior disorder; MSA, multiple system atrophy; MSA-P, MSA-parkinsonian type; MSA-C, MSA-cerebellar type; PSP, progressive supranuclear palsy; HC, healthy control.

**Fig. S2** 3D gradient-echo T2* images (echo 1-echo 4) for a representative case from each patient and healthy control groups


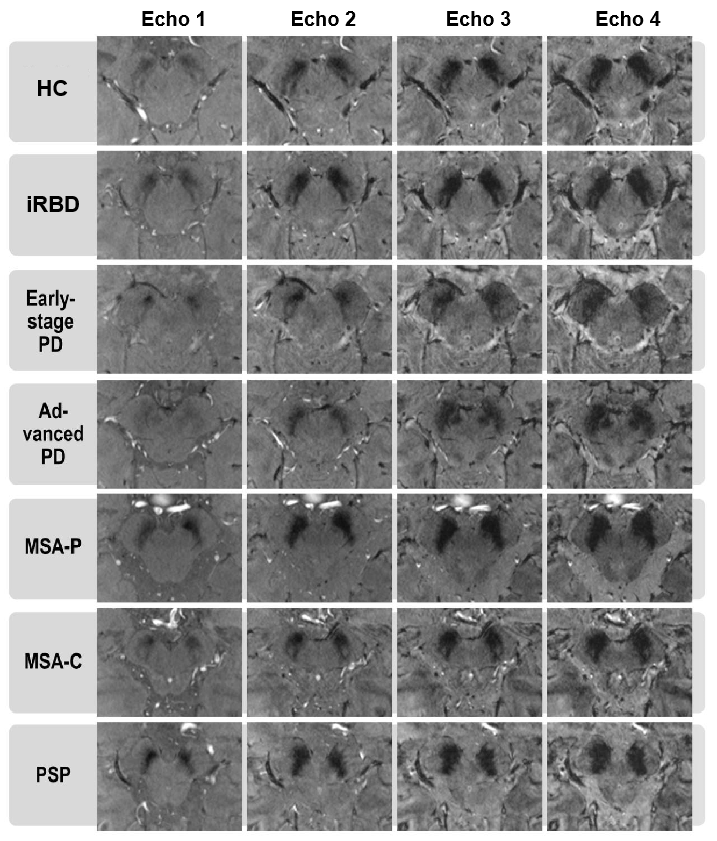


3D, three-dimensional; PD, Parkinson’s disease; iRBD, idiopathic rapid eye movement sleep behavior disorder; MSA, multiple system atrophy; MSA-P, MSA-parkinsonian type; MSA-C, MSA-cerebellar type; PSP, progressive supranuclear palsy; HC, healthy control.

**Fig. S3** ROC curves for the three DNH rating scales at echo 2 in the pooled dataset


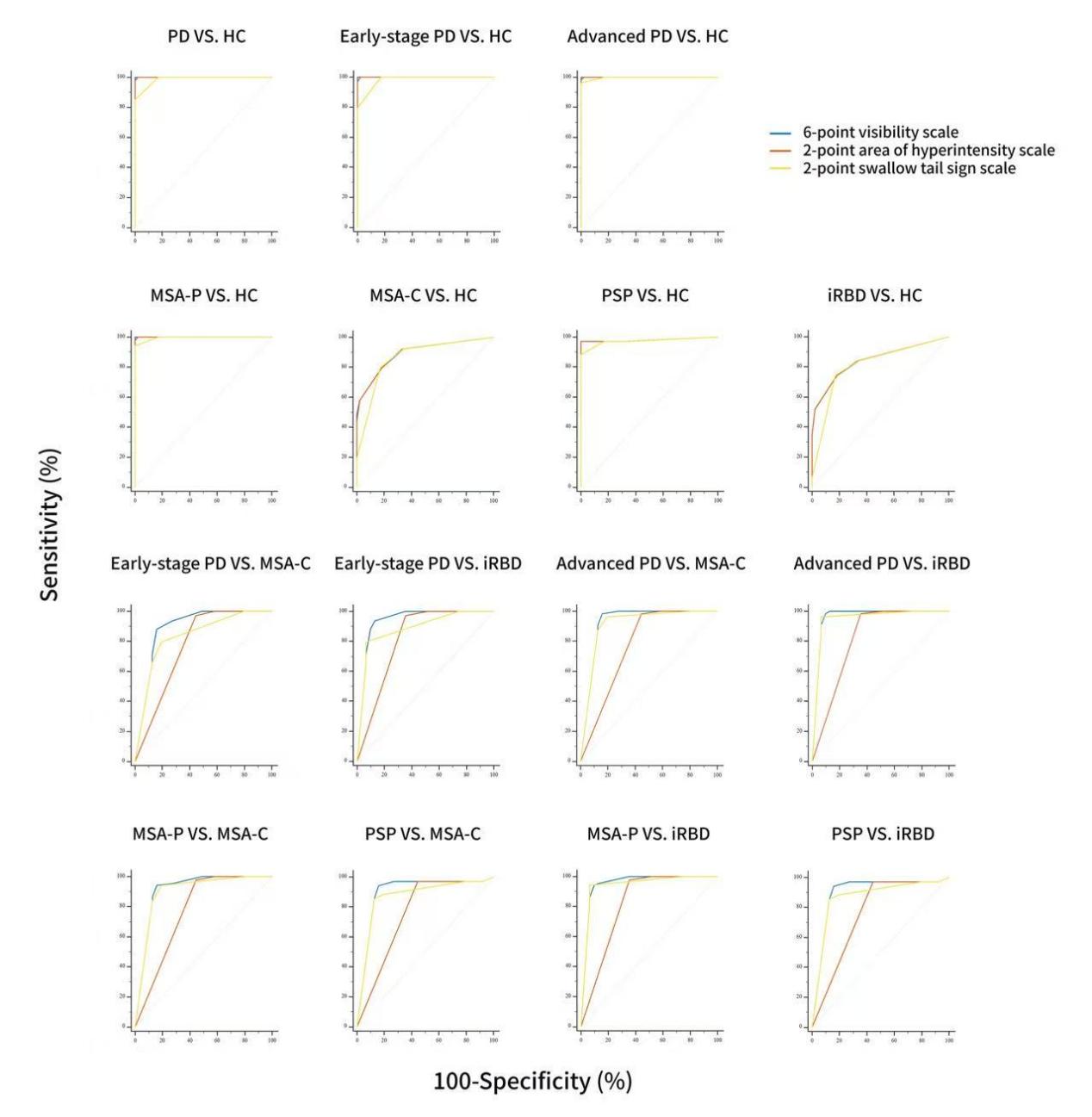


ROC, receiver operating curve; DNH, dorsal nigral hyperintensity; PD, Parkinson’s disease; iRBD, idiopathic rapid eye movement sleep behavior disorder; MSA, multiple system atrophy; MSA-P, MSA-parkinsonian type; MSA-C, MSA-cerebellar type; PSP, progressive supranuclear palsy; HC, healthy control.

**Fig. S4** Receiver operating characteristic curves for the optimal DNH rating scale in the development cohort, its performance in the validation cohort, and its reassessment in the pooled dataset

**
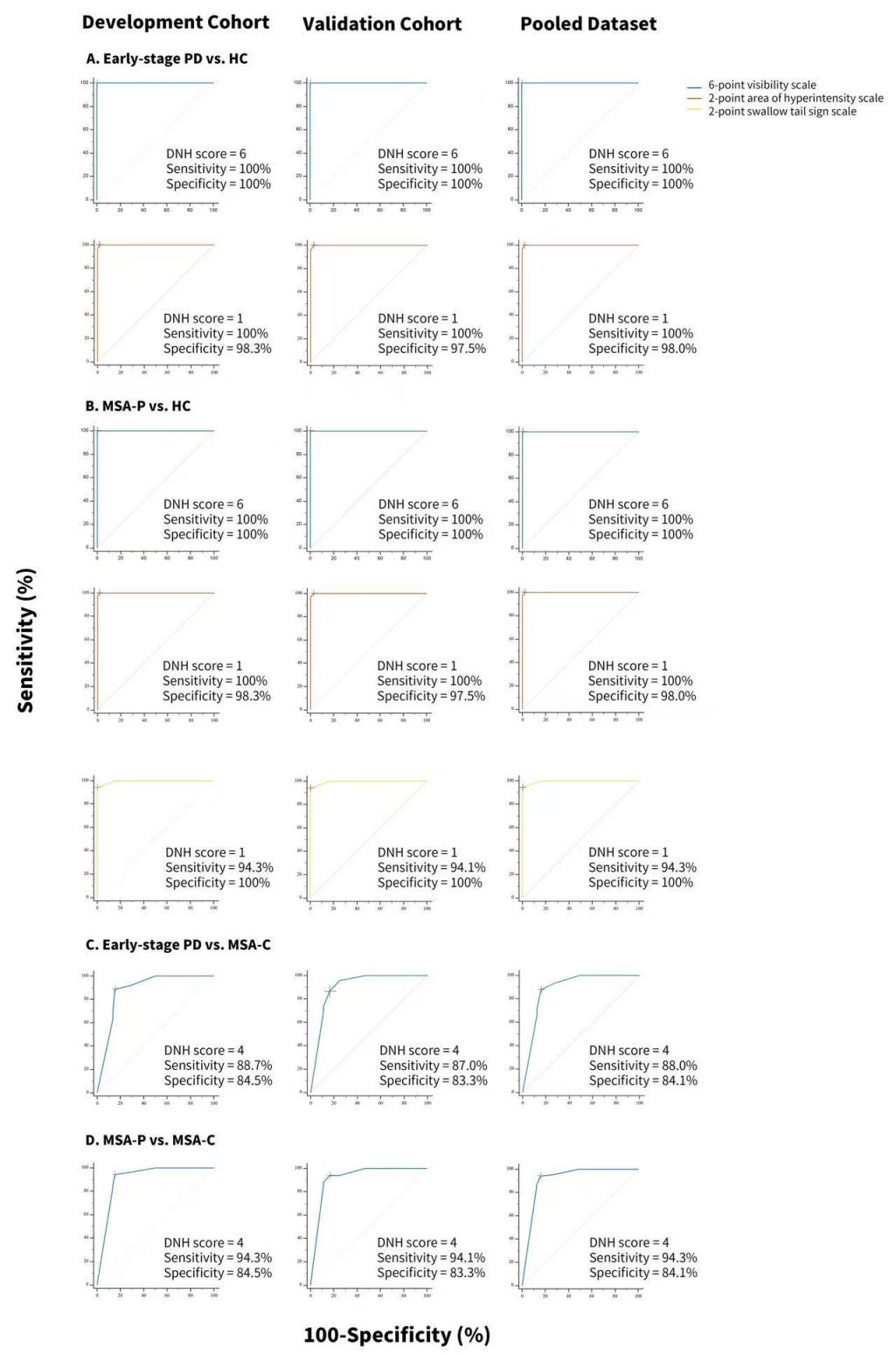
**

PD, Parkinson’s disease; MSA, multiple system atrophy; MSA-P, MSA-parkinsonian type; MSA-C, MSA-cerebellar type; HC, healthy control; DNH, dorsal nigral hyperintensity.

**Analysis S1 Comparison of participant characteristics**

**Development Cohort**

In the multi-group comparison among early-stage PD, advanced PD, iRBD, MSA-P, MSA-C, PSP, and HC groups, advanced PD and PSP were significantly older than early-stage PD, MSA-P, and MSA-C patients (*P* < 0.05). PSP patients were also significantly older than HC (*P* < 0.001). No significant difference in sex was found among all patient and HC groups (*p* = 0.137). MSA-P, MSA-C, and PSP patients had less years of education compared with HC (*P* < 0.05). The advanced PD group had significantly longer disease duration compared with the early-stage PD, iRBD, MSA-P, MSA-C, and PSP groups (*P* < 0.001). Early-stage PD and MSA-C patients had significantly lower MDS-UPDRS III scores compared with advanced PD, MSA-P, and PSP patients (*P* < 0.05), while iRBD patients had significantly lower MDS-UPDRS III scores compared with early-stage PD, advanced PD, MSA-P, MSA-C, and PSP patients (*P* < 0.001). Early-stage PD patients had significantly lower H-Y stage compared with advanced PD patients (*P* < 0.001). PSP patients had lower MMSE scores compared with patients with early-stage PD, advanced PD, iRBD, MSA-P, and MSA-C as well as HC (*P* < 0.05). Similarly, PSP patients had lower MoCA scores than patients with early-stage PD, advanced PD, iRBD, and MSA-P as well as HC (*P* < 0.05). The early-stage PD, advanced PD, and MSA-P groups had higher HAMA scores compared with HC (*P* < 0.05), while PSP and advanced PD patients showed higher HAMD scores compared with HC (*P* < 0.05).

**Validation Cohort**

No significant differences in age (*p* = 0.344), sex (*p* = 0.314), and years of education (*p* = 0.145) were found among early-stage PD, MSA-P, MSA-C, and HC. No significant difference in disease duration was found among early-stage PD, MSA-P, and MSA-C (*p* = 0.520). MSA-P patients had significantly higher MDS-UPDRS III scores compared with early-stage PD and MSA-C patients (*P* < 0.001). No significant differences in MMSE and MoCA scores were found among early-stage PD, MSA-P, MSA-C, and HC (*p* = 0.052 and *p* = 0.117, respectively). MSA-P, and MSA-C patients had significantly higher HAMA scores (*P* < 0.05) than HC, and early-stage PD, MSA-P, and MSA-C patients had significantly higher HAMD scores (*P* < 0.05) than HC.

**Development Cohort vs. Validation Cohort**

Comparisons between early-stage PD, MSA-P, MSA-C, and HC groups in the development and validation cohorts revealed no significant differences in demographic characteristics including age, sex, and years of education. MSA-C patients in the development cohort had significantly lower HAMA scores than MSA-C patients in the validation cohort (*p* = 0.010). No significant differences in other clinical characteristics were observed in the early-stage PD, MSA-P, MSA-C, and HC groups.

**Pooled Dataset**

No significant differences in age (*p* = 0.816), sex (*p* = 0.088), and years of education (*p* = 0.170) were found between PD and HC. PD patients had significantly lower MMSE scores (*p* = 0.034), lower MoCA scores (*p* = 0.020), higher HAMA scores (*P* < 0.001), and higher HAMD scores (*P* < 0.001) than HC.

In the multi-group comparison among early-stage PD, advanced PD, iRBD, MSA-P, MSA-C, PSP, and HC groups, advanced PD and PSP were significantly older than early-stage PD, MSA-P, MSA-C, and HC (*P* < 0.05). No significant difference in sex was found among all patients and HC groups (*p* = 0.054). MSA-P, MSA-C, and PSP patients had less years of education compared with HC (*P* < 0.05). The advanced PD group had significantly longer disease duration compared with the early-stage PD, iRBD, MSA-P, MSA-C, and PSP groups (*P* < 0.001). Early-stage PD and MSA-C patients had significantly lower MDS-UPDRS III scores compared with advanced PD, MSA-P, and PSP patients (*P* < 0.05), and iRBD patients had significantly lower MDS-UPDRS III scores compared with early-stage PD, advanced PD, MSA-P, MSA-C, and PSP patients (*P* < 0.001). Early-stage PD had significantly lower H-Y stage compared with advanced PD (*P* < 0.001). MSA-P had lower MMSE scores than HC (*p* = 0.005). MSA-C patients showed lower MMSE as well as MoCA scores than HC (*P* < 0.05). PSP patients had lower MMSE and MoCA scores compared with early-stage PD, advanced PD, iRBD, MSA-P, MSA-C, and HC (*P* < 0.05). The early-stage PD, advanced PD, MSA-P, and MSA-C groups had higher HAMA scores compared with HC (*P* < 0.05), while early-stage PD, MSA-P, MSA-C, and PSP patients showed higher HAMD scores compared with HC (*P* < 0.05).

**Analysis S2 Comparison of diagnostic and differential diagnostic performance between T2* echoes**

**Development Cohort**

We compared the diagnostic and differential diagnostic accuracy of the four dichotomous DNH assessment methods across the four T2* echoes. Echo 1 and echo 2 demonstrated significantly higher diagnostic accuracy than echo 4 when comparing between early-stage PD, advanced PD, and MSA-P and HC using the bilaterally normal and unilaterally normal methods. Additionally, echo 1 and echo 2 showed significantly higher diagnostic accuracy than echo 4 in the comparisons between MSA-C and HC as well as PSP and HC using the unilaterally normal method,

Comparisons of AUC of the three DNH rating scales in disease diagnosis and differential diagnosis were also performed. Using the 6-point visibility scale, echo 2 showed a significantly higher AUC than echo 4 in distinguishing MSA-C from HC, and in differentiating early-stage PD, advanced PD, MSA-P, and PSP from MSA-C. Using the 2-point area of hyperintensity scale, echo 2 demonstrated a higher AUC than other echoes for diagnosing all disease groups compared with HC, as well as for differentiating early-stage PD, advanced PD, and MSA-P from iRBD than echo 3. As for the 2-point swallow tail sign scale, echo 2 showed a higher AUC than other echoes for diagnosing early-stage PD and iRBD compared with HC, and for the differentiating early-stage PD, advanced PD, MSA-P, and PSP from MSA-C.

**Pooled Dataset**

Echo 4 demonstrated significantly higher diagnostic accuracy than echoes 1 and 2 for diagnosing PD using the bilaterally detected and unilaterally detected methods, as well as for diagnosing early-stage PD using the bilaterally detected method. Echo 2, however, showed significantly higher diagnostic accuracy than the other echoes when comparing between all disease groups (PD, early-stage PD, advanced PD, iRBD, MSA-P, MSA-C, and PSP) and HC using to the bilaterally normal and the unilaterally normal methods.

According to the 6-point visibility scale, echo 2 demonstrated significantly a higher AUC than the other echoes in distinguishing MSA-C from HC, and in differentiating early-stage PD, advanced PD, MSA-P, and PSP from MSA-C. Furthermore, using the 2-point area of hyperintensity scale, echo 2 showed a higher AUC than the other echoes for: (1) diagnosing all disease groups compared with HC, (2) differentiating early-stage PD, advanced PD, and MSA-P from MSA-C and iRBD, and (3) differentiating PSP from MSA-C. Using the 2-point swallow tail sign scale, echo 2 demonstrated a higher AUC than the other echoes for diagnosing PD, early-stage PD, MSA-P, and MSA-C compared with HC, as well as for differentiating early-stage PD, advanced PD, MSA-P, and PSP from MSA-C.

**Analysis S3 Correlation analysis between clinical characteristics and DNH scores in PD and MSA-C patients**

Correlation analysis was performed between DNH rating scale scores and clinical characteristics in 44 PD and 76 MSA-C patients with detectable DNH. For PD patients with detectable DNH, the scores of three DNH rating scales (6-point visibility scale, 2-point area of hyperintensity scale, and 2-point swallow tail sign scale) did not correlate with disease duration (*p* = 0.150, *p* = 0.460, *p* = 0.098, respectively), MDS-UPDRS III scores (*p* = 0.260, *p* = 0.716, *p* = 0.224, respectively), or H-Y stages (*p* = 0.828, *p* = 0.387, *p* = 0.650, respectively). For MSA-C patients with detectable DNH, the scores of three DNH rating scales did not correlate with disease duration (*p* = 0.833, *p* = 0.711, *p* = 0.701, respectively) or MDS-UPDRS III scores (*p* = 0.251, *p* = 0.185, *p* = 0.937, respectively).

**Analysis S4 Comparison of clinical characteristics in MSA-P and PSP patients regarding the detectability of the DNH**

Using the pooled dataset, we compared the age at onset and disease duration between patients with and without bilateral detectable DNH in the MSA-P and PSP groups. In the MSA-P group, patients with bilateral detectable DNH had a significantly shorter disease duration (*p* = 0.013), with no significant difference in age at onset (*p* = 0.192) compared with patients without bilateral detectable DNH. In the PSP group, no significant differences were found in either age at onset (*p* = 0.629) or disease duration (*p* = 0.624) between patients with and without bilateral detectable DNH.

**References**

1. Pipe JG. Motion correction with PROPELLER MRI: Application to head motion and free-breathing cardiac imaging. Magn Reson Med. 1999;42(5):963–9.

2. Blazejewska AI, Schwarz ST, Pitiot A, Stephenson MC, Lowe J, Bajaj N, et al. Visualization of nigrosome 1 and its loss in PD: Pathoanatomical correlation and in vivo 7 T MRI. Neurology. 2013;81(6):534–40.

3. Kim JM, Jeong HJ, Bae YJ, Park SY, Kim E, Kang SY, et al. Loss of substantia nigra hyperintensity on 7 Tesla MRI of Parkinson’s disease, multiple system atrophy, and progressive supranuclear palsy. Parkinsonism & Related Disorders. 2016;26:47–54.

4. Schwarz ST, Mougin O, Xing Y, Blazejewska A, Bajaj N, Auer DP, et al. Parkinson’s disease related signal change in the nigrosomes 1–5 and the substantia nigra using T2* weighted 7T MRI. NeuroImage: Clinical. 2018;19:683–9.

5. Noh Y, Sung YH, Lee J, Kim EY. Nigrosome 1 Detection at 3T MRI for the Diagnosis of Early-Stage Idiopathic Parkinson Disease: Assessment of Diagnostic Accuracy and Agreement on Imaging Asymmetry and Clinical Laterality. AJNR Am J Neuroradiol. 2015;36(11):2010–6.

6. Liu X, Wang N, Chen C, Wu PY, Piao S, Geng D, et al. Swallow tail sign on susceptibility map-weighted imaging (SMWI) for disease diagnosing and severity evaluating in parkinsonism. Acta Radiol. 2021;62(2):234–42.

7. Wang N, Liu X, Li L, Zuo C, Wang J, Wu P, et al. Screening for Early‐Stage Parkinson’s Disease: Swallow Tail Sign on MRI Susceptibility Map‐Weighted Images Compared With PET. Journal of Magnetic Resonance Imaging : JMRI. 2021;53(3):722-30.

8. Gramsch C, Reuter I, Kraff O, Quick HH, Tanislav C, Roessler F, et al. Nigrosome 1 visibility at susceptibility weighted 7T MRI—A dependable diagnostic marker for Parkinson’s disease or merely an inconsistent, age-dependent imaging finding? PLoS ONE. 2017;12(10):e0185489.

9. Avants B, Epstein C, Grossman M, Gee J. Symmetric diffeomorphic image registration with cross-correlation: Evaluating automated labeling of elderly and neurodegenerative brain. Medical Image Analysis. 2008;12(1):26–41.

10. Chan KS, Marques JP. SEPIA—Susceptibility mapping pipeline tool for phase images. NeuroImage. 2021;227:117611.

11. Forstmann BU, Keuken MC, Schafer A, Bazin PL, Alkemade A, Turner R. Multi-modal ultra-high resolution structural 7-Tesla MRI data repository. Sci Data. 2014;1(1):140050.

12. Bozdogan H. Akaike’s Information Criterion and Recent Developments in Information Complexity. Journal of Mathematical Psychology. 2000;44(1):62–91.

13. Nisbet R, Miner G, Yale K, Elder JF, Peterson AF. Handbook of statistical analysis and data mining applications. Second edition. ed. London: Academic Press; 2018.

14. Vaishnavi SN, Vlassenko AG, Rundle MM, Snyder AZ, Mintun MA, Raichle ME. Regional aerobic glycolysis in the human brain. Proc Natl Acad Sci USA. 2010;107(41):17757–62.

15. Stebbins GT, Goetz CG, Burn DJ, Jankovic J, Khoo TK, Tilley BC. How to identify tremor dominant and postural instability/gait difficulty groups with the movement disorder society unified Parkinson’s disease rating scale: Comparison with the unified Parkinson’s disease rating scale. Movement Disorders. 2013;28(5):668–70.
